# The Cellulosome Paradigm in An Extreme Alkaline Environment

**DOI:** 10.3390/microorganisms7090347

**Published:** 2019-09-12

**Authors:** Paripok Phitsuwan, Sarah Moraïs, Bareket Dassa, Bernard Henrissat, Edward A. Bayer

**Affiliations:** 1 Department of Biomolecular Sciences, The Weizmann Institute of Science, Rehovot 7610001, Israel; 2 Faculty of Natural Sciences, Ben-Gurion University of the Negev, Beer-Sheva 8499000, Israel; 3 Architecture et Fonction des Macromolécules Biologiques, CNRS and Aix-Marseille University and CNRS, 13288 Marseille, France; 4 USC1408, INRA, Architecture et Fonction des Macromolécules Biologiques, 13288 Marseille, France

**Keywords:** glycoside hydrolases, *Clostridium alkalicellulosi*, alkaliphilic bacterium, cohesin, dockerin, scaffoldin, biomass degradation

## Abstract

Rapid decomposition of plant biomass in soda lakes is associated with microbial activity of anaerobic cellulose-degrading communities. The alkaliphilic bacterium, *Clostridium alkalicellulosi*, is the single known isolate from a soda lake that demonstrates cellulolytic activity. This microorganism secretes cellulolytic enzymes that degrade cellulose under anaerobic and alkaliphilic conditions. A previous study indicated that the protein fraction of cellulose-grown cultures showed similarities in composition and size to known components of the archetypical cellulosome *Clostridium thermocellum*. Bioinformatic analysis of the *C. alkalicellulosi* draft genome sequence revealed 44 cohesins, organized into 22 different scaffoldins, and 142 dockerin-containing proteins. The modular organization of the scaffoldins shared similarities to those of *C. thermocellum* and *Acetivibrio cellulolyticus*, whereas some exhibited unconventional arrangements containing peptidases and oxidative enzymes. The binding interactions among cohesins and dockerins assessed by ELISA, revealed a complex network of cellulosome assemblies and suggested both cell-associated and cell-free systems. Based on these interactions, *C. alkalicellulosi* cellulosomal systems have the genetic potential to create elaborate complexes, which could integrate up to 105 enzymatic subunits. The alkalistable *C. alkalicellulosi* cellulosomal systems and their enzymes would be amenable to biotechnological processes, such as treatment of lignocellulosic biomass following prior alkaline pretreatment.

## 1. Introduction

During the past decade, we have experienced increasing interest in the development of new technologies for alternative energy production using renewable and sustainable sources with beneficial environmental impact. Owing to the potential for replacing fossil-based resources in terms of availability, renewability, and costs, lignocellulosic biomass is now considered a suitable feedstock for conversion of fermentable sugars to biofuels, such as ethanol [1]. However, because of the natural recalcitrance of the plant cell walls to unlock these sugars, harnessing this alternative energy source remains challenging [2,3].

In nature, cellulolytic microorganisms use different strategies to degrade plant cell walls in order to gain their preferred metabolizable sugars [4]. Among the enzymatic paradigms, the “cellulosome”, a discrete multi-enzyme complex comprising carbohydrate-active enzymes, is considered one of the most efficient systems for deconstruction of cellulosic biomass [5]. The cellulosome is produced by selected anaerobic microorganisms and is particularly prevalent in clostridial species [6,7]. It was first discovered in the thermophilic, anaerobic bacterium, *Clostridium thermocellum* [8]. Extensive studies on the *C. thermocellum* cellulosome revealed that it comprises a high-molecular-weight “scaffoldin” subunit, composed of several type I cohesin modules, a type II dockerin module and a carbohydrate-binding module (CBM), interlinked by flexible proline/threonine-rich linker segments [9]. Each cellulosomal enzyme contains a type I dockerin module that interacts with complimentary type I cohesins via a high-affinity interaction, which integrates them into the complex [10,11]. The cellulosome is attached to the bacterial cell surface through a second cohesin–dockerin interaction (type II), in which the dockerin on the primary scaffoldin binds to cohesin(s) of anchoring scaffoldins, attached to the cell surface by virtue of surface-layer homology (SLH) modules [9]. The primary scaffoldin targets the entire set of cellulosomal components and the parent bacterial cells to the cellulosic substrate via its CBM [6]. The latter targeting effect and proximity of the cellulosomal enzyme subunits enhance the synergistic interaction among the components, and together represent key factors for effective degradation of the recalcitrant cellulosic substrate [12,13].

More elaborate cellulosomal systems have been described in *Ruminococcus flavefaciens* [14,15], *Acetivibrio cellulolyticus* [16,17], *Clostridium clariflavum* [18], *Herbivorax saccinola* [19,20], and (*Pseudo*)*Bacteroides cellulosolvens* [21,22,23]. These cellulosome-producing bacteria secrete several types of scaffoldins to form elaborate complex networks, in order to increase the number of enzymatic subunits and/or integrate specific proteins into the complex. The diversity and versatility of the cellulosomes may allow the microorganisms to adapt to different environments, particularly during growth [9,24]. In *C. clariflavum*, one of the predicted cellulosomal complexes consists of an anchoring scaffoldin containing four type II cohesin modules interacting with an adaptor scaffoldin containing five type II cohesin modules that would interact specifically with a primary scaffoldin that carries eight enzymatic subunits. At full capacity, this single complex would theoretically integrate 160 enzyme subunits and comprises the largest cellulosomal complex described to date [18]. The *B. cellulosolvens* genome encodes for 32 cohesin-containing scaffoldin proteins and 212 dockerin-containing genes, which makes this bacterium the most elaborate and comprehensive cellulosome-producing microorganism [22,23].

The natural microorganisms’ habitats may impact their microbial features, evolution, and adaptations to their specific ecosystem. Most of the cellulosome-producing bacteria to date have been isolated from environments of neutral pH [25], while cellulolytic microorganisms growing at high pH environments are rarely found [19]. The soda lake is an interesting source to search for potential cellulosome-producing bacteria, since it was observed that plant and algal debris accumulated in the soda lakes are actively decomposed by the anaerobic microbial community [26,27]. This community is potentially populated by several cellulose-degrading microorganisms with distinct cellulase systems and properties that resist the extreme environmental conditions, i.e., high salt and high pH [27,28]. In this context, *Clostridium alkalicellulosi* DSM17461^T^ was isolated from the Verkhnee Boloe soda lake (pH ~10) and was found to be an anaerobic alkaliphilic cellulolytic bacterium [27,28]. The bacterium is Gram-positive, spore-forming, and rod-shaped, which grows from pH 8.0 to 10.2 with an optimal growth at pH 9.0. The bacterium is able to utilize cellulose, xylan, and natural biopolymers, including matgrass (*Nardus stricta*) stem and algal *Cladophora sivashensis* biomass [27]. Taxonomic analysis based on 16S rRNA gene sequences revealed that *C. alkalicellulosi* is classified among cellulolytic clostridia of cluster III, and its closest relative is *C. thermocellum*, followed by *Clostridium aldrichii* and *A. cellulolyticus*. A study by Zvereva et al. [28] demonstrated the potential of this strain to produce cellulosomes. A protein preparation from the growth culture of *C. alkalicellulosi* exhibited some similarities in terms of size and subunit compositions to the protein components of the *C. thermocellum* cellulosome [28]. However, the identity of the individual protein components and their genes were not determined, due to the lack of the available genomic sequence at the time of the study.

In the present work, we employed computational tools to reveal the genes encoding the cellulosomal components (cohesin and dockerin modules) in the *C. alkalicellulosi* genome. Using this approach, we successfully identified several dockerin-bearing proteins and cohesin-bearing scaffoldins in the genome of this bacterium. The predicted proteins were compared with the known cellulosomal components of *C. thermocellum* and *A. cellulolyticus*. The cohesin–dockerin interaction was assessed for selected putative cohesin and dockerin modules of *C. alkalicellulosi* by affinity-based ELISA of the recombinant proteins. Their binding interaction profiles were used to predict potential assemblies of the cellulosomes in *C. alkalicellulosi*. This work provides a comprehensive investigation that confirms the existence of the cellulosome system in *C. alkalicellulosi* and extends our knowledge of its features from an alkaline environment.

## 2. Materials and Methods

### 2.1. Genomic DNA and Genome Sequence Availability

The genomic DNA of *C. alkalicellulosi* DSM17461^T^ was purchased from the German Collection of Microorganisms and Cell Cultures (DSMZ, Braunschweig, Germany). Genome sequences of *C. alkalicellulosi* DSM17461^T^ (JGI Gold project ID: Ga0025046), *A. cellulolyticus* CD2 (NZ AEDB02000000), and *C. thermocellum* ATCC 27405 (CP000568) were retrieved from the GenBank of NCBI [29].

### 2.2. Identification of Cohesins and Dockerins in the Bacterial Genome

To predict putative cohesins and dockerins, the DNA contigs of *C. alkalicellulosi* DSM17461^T^ were analyzed by BLAST algorithm searches [30], using sequences of known cohesin and dockerin modules as queries. Hits of E-value lower than 10^−4^ were retrieved and checked individually by examining their sequence characteristics. For instance, dockerin modules were expected to contain two Ca^+2^ binding repeats, putative helices, and linker regions. Low-score hits of dockerins and cohesins were analyzed by comparing them against known dockerin or cohesin sequences, respectively (http://www.weizmann.ac.il/Biomolecular_Sciences/Bayer/sites/Biomolecular_Sciences.Bayer/files/uploads/dockerins_setup.txt and http://www.weizmann.ac.il/Biomolecular_Sciences/Bayer/sites/Biomolecular_Sciences.Bayer/files/uploads/cohesins_setup.txt). Multiple sequence alignment of cohesins and dockerins were performed using ClustalO [31], with manual edits if required. Signal peptide sequences were predicted using the SignalP versions 3.0 or 4.1 server [32]. Sequence logos of dockerins were created using Weblogo v.2.8.2 [33].

### 2.3. Annotation of Dockerin-Containing Enzymes

To identify the catalytic modules, dockerin-containing proteins of *C. alkalicellulosi* DSM17461^T^ were bioinformatically determined and annotated using the CAZy database [34,35]. This enabled the identification of the catalytic modules and their family classification, based on sequence conservation, for glycoside hydrolases, carbohydrate esterases, polysaccharide lyases, carbohydrate-binding modules, and glycosyltransferases. The proteins were further analyzed for additional conserved domains using the CD-search website [36] and the Pfam database [37].

### 2.4. Phylogenetic Analysis

Multiple sequence alignments of cohesins were conducted using ClustalO [31]. Phylogenetic trees were constructed using the robust phylogenetic analysis tool [38]. The sequences were analyzed, and the trees were built with optimization through the default bootstrap under the “One Click” mode option. Then, the tree was manually edited for improved visualization using iTOL v.3 [39].

### 2.5. Construction of CBM3a-Fused Cohesins

The pET28a plasmid was used to create fusion proteins CBM3a-Cohesin (CBM-Coh). The gene encoding the CBM3a of *C. thermocellum* scaffoldin CipA [40] was inserted into the pET28a plasmid at NcoI and BamHI sites as previously described by Barak et al. 2005 [41]. The genes encoding target cohesin modules were designed to have BamHI and XhoI restriction sites and amplified by specific PCR primers using the genomic DNA of *C. alkalicellulosi* DSM17461^T^ as a template. The PCR products were purified by the HiYield gel-PCR fragment extraction kit (Real Biotech Corporation, RBC, Banqiao City, Taiwan) and were double digested by BamHI (5′ terminus) and XhoI (3′ terminus) FastDigest enzymes (Thermo scientific, Fermentas UAB, Vilnius, Lithuania). Then, the resultant DNA fragments were ligated into the pET28a-CBM3a cassette [41,42]. The plasmids were transformed into an *Escherichia coli* DH5α strain and purified via QIAprep spin miniprep kit (QIAGEN GmbH, D-40724 Hilden, Germany). The sequence identity was confirmed by DNA sequencing.

### 2.6. Xylanase-Dockerin (Xyn-Doc) Cassettes

The cloning methodology of the Xyn-Doc construction was conducted similar to the CBM-Coh cassette, with minor modifications. A PCR product of *G. stearothermophilus* T6 xylanase with a His-tag and BspHI (5′ terminus) and KpnI (3′ terminus) restriction sites was obtained [41,42,43] and inserted into the pET9d vector. The genes encoding dockerins of interest were amplified using specific primers (Supplementary Table S1) by PCR with addition of the KpnI site at the 5′ terminus and the BamHI at the 3′ terminus. The amplified dockerin-encoding genes were double digested and subcloned into the KpnI and BamHI sites of the pET9d-Xyn cassette to form a plasmid pET9d-Xyn-Doc.

### 2.7. Protein Expression

The pET28a cassette containing the CBM-Coh fusion proteins and the pET9d cassette containing the Xyn-Doc fusion proteins were transformed into *E. coli* BL21 (DE3) strains and plated onto Luria-Bertani (LB) agar plates supplemented with kanamycin (50 mg/L; Sigma-Aldrich, Rehovot, Israel). For each plate, 5 mL of LB broth were added to remove the bacterial colonies on the agar surfaces and resuspend the cells. The cell suspensions were then added to 1 L of LB supplemented with 50 mg/L kanamycin and 2 mM CaCl_2_ (final concentrations) and were grown for up to 2.5 h at 37 °C to reach an OD_600_ of 0.8 to 1.0. The expression of proteins was induced by adding isopropyl-1-thio-β-D-galactoside (IPTG; Fermentas UAB, Vilnius, Lithuania) at a final concentration of 0.2 mM, and the culture was grown continually at 16 °C for 16 h. The cells were harvested by centrifugation at 4250× *g* at 4 °C for 15 min.

### 2.8. CBM-Coh Purification

For the purification of the recombinant CBM-Coh, the cells after centrifugation were resuspended in 30 mL TBS (tris-buffered saline, 137 mM NaCl, 2.7 mM KCL, and 25 mM Tris-HCl, pH 7.4), and protease-inhibitor cocktail (1 mM phenylmethylsulfonyl fluoride, 0.4 mM benzamidine, and 0.06 mM benzamide) was added to prevent protein degradation. The cells were sonicated on ice and centrifuged at 20,200× *g*, 4 °C for 30 min to remove the cell debris. The cell-free extract was then added to 2 g of microporous bead cellulose preswollen gel (IONTO- SORB, Ustinad Labem, Czech Republic) and incubated for 1 h, with a gentle rotation, at 4 °C. The mixture was then loaded onto a gravity column and washed with 100 mL of TBS containing 1 M NaCl, followed by 100 mL of TBS. The proteins were then eluted with three 10-mL portions of elution buffer (1% (v/v) triethanolamine (TEA) in TBS). The three fractions were subjected to SDS-PAGE in order to analyze the molecular mass and homogeneity of the recombinant protein. Then the protein was dialyzed against TBS containing 5 mM CaCl_2_ at 4 °C overnight.

### 2.9. Xyn-Doc Purification

After centrifugation, the cells were resuspended in 30 mL of TBS supplemented with 5 mM imidazole and protease-inhibitor cocktail. Cells were disrupted by sonication on ice and centrifuged at 20,200× *g*, 4 °C for 30 min. The purification was performed in a batch Ni-NTA purification system as described previously by Vazana et al. 2010 [13]. The eluted proteins were collected at a fraction volume of 2 mL. The molecular mass and purity of the purified proteins were analyzed by SDS-PAGE. The fractions containing the protein of interest were collected and pooled, and they were dialyzed at 4 °C overnight against TBS containing 5 mM CaCl_2_.

### 2.10. Protein Concentration and Storage

Protein concentrations were measured by absorbance at 280 nm, and the concentrations were determined using the molar extinction coefficients derived from the known composition of amino-acids of each protein sequence. The molar extinction coefficients were calculated using the ExPASy ProtParam tool [44]. The proteins were concentrated by Amicon ultra concentrators (Millipore, Carrigtwohill, Co. Cork, Ireland), and stored at −20 °C in the presence of 50% (v/v) glycerol.

### 2.11. ELISA

Affinity-based ELISA was performed as described previously by Barak et al. 2005 [41]. The 96-well ELISA plates (Nunc, A/S, Roskilde, Denmark) were coated with the fusion proteins Xyn-Docs (or CBM-Cohs) at a concentration of 1 μg/mL, and different concentrations of CBM-Cohs (or Xyn-Docs) ranging from 0.001 to 1000 ng/mL were used to detect specific cohesin–dockerin interactions. The interactions were examined immunochemically by using anti-CBM primary antibody (or anti-xylanase primary antibody) and horseradish peroxidase (HRP)-labeled secondary antibody in the presence of chromogenic substrate TMB (3,3′,5,5′-Tetramethylbenzidine, Dako, Denmark). The reaction product (color formation) was measured at the absorbance of 450 nm. The reference concentration of a CBM-Coh (or Xyn-Doc) standard that generates a maximum response was employed for comparison of the level of response produced by other test Xyn-Doc constructs at that concentration.

## 3. Results

### 3.1. Genomic Analysis Reveals Cohesin- and Dockerin-Containing Proteins

**Figure 1:**
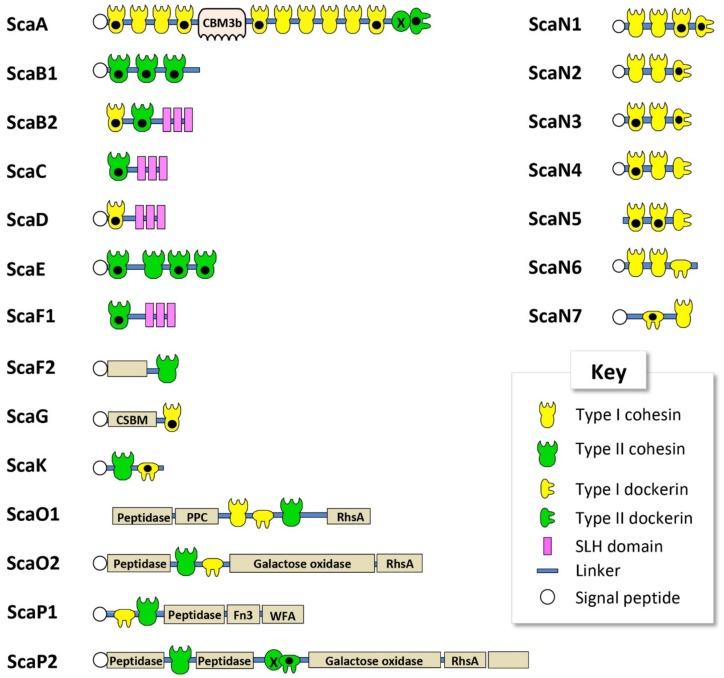
Schematic representation of the cohesin-containing proteins, identified bioinformatically in the *Clostridium alkalicellulosi* draft genome. Black dots indicate cohesin and dockerin modules of the designated scaffoldins that were expressed and examined for specific interactions in the current study. Definitions for the abbreviations and GI accession numbers for the scaffoldins are available in Table 1.

To reveal components of the cellulosomal systems in *C. alkalicellulosi* DSM17416, bioinformatic analyses of the genome sequence were performed. The size of the *C. alkalicellulosi* genome was 5.3 Mb. *C. alkalicellulosi* had the highest number of GH9 cellulases and CBM modules encoded in its genome, compared to other cellulolytic mesophiles. In total, 142 putative dockerin-containing proteins and 45 putative cohesin-containing proteins were identified in the draft genome sequence of this bacterium. Based on sequence similarity [16], the dockerin sequences were divided into two groups: Type I (138 modules) and type II (four modules; Supplementary Table S2). The cohesin sequences were also divided into two groups: Type I (31 modules) and type II (14 modules; Supplementary Table S3). The modular organization of the cohesins into the molecular scaffoldins is shown in Figure 1. The nomenclature of the scaffoldins from ScaA to ScaN followed the previously established nomenclature of characterized cellulosomal species of *C. thermocellum*, *A. cellulolyticus*, *C. clariflavum*, and *B. cellulosolvens* [17,18,22,45], guided by the modular architecture of the given scaffoldin and/or the phylogenetic relationship of its cohesin(s) (Table 1).

### 3.2. Cohesin Modules are Diverse and Similar to C. thermocellum Cohesins

**Figure 2:**
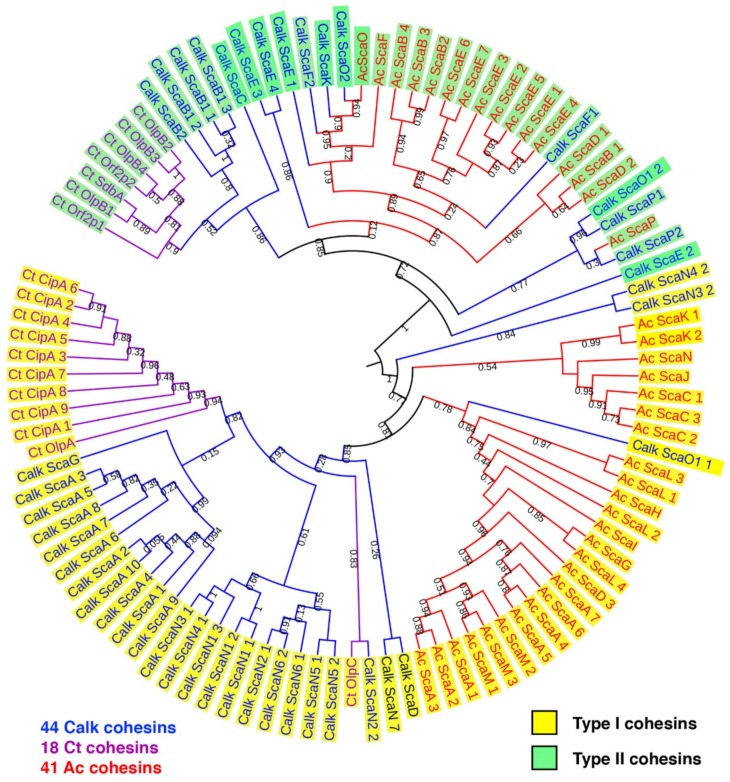
Phylogenetic relationship of *C. alkalicellulosi* cohesins with other characterized cohesins from *C. thermocellum* and *A. cellulolyticus*. The tree was constructed based on 44, 18, and 41 cohesin modules of *C. alkalicellulosi* (Calk, blue), *C. thermocellum* (Ct, purple), and *A. cellulolyticus* (Ac, red), respectively. Numbers on the branches represent bootstrap values.

The phylogenetic relationship of the *C. alkalicellulosi* cohesins was performed with related strains and to determine the extent of their diversity. The cohesin sequences from the two closest cellulosome-producing relatives, namely *C. thermocellum* and *A. cellulolyticus*, were selected and used as references. The phylogenetic tree showed that all type I and II cohesins were affiliated with the respective cluster of *C. thermocellum* and/or *A. cellulolyticus* cohesins (Figure 2).

### 3.3. Cohesin-Containing Scaffoldins Exhibit Conventional and Unconventional Modular Organizations

All the *C. alkalicellulosi* scaffoldins, except ScaB2, ScaC, ScaF1, ScaN5, and ScaO1, contained a signal peptide at their N-terminus, indicating their secretion outside the cells (Figure 1, Table 1). As it is doubtful that these very large scaffoldins would function inside the bacterial cell (especially ScaB2 and ScaF1, which contained SLH modules), the absence of signal peptides for these three scaffoldins could stem from the limitation of the detection program or sequencing/assembly errors in that particular region of the draft genome sequence. In general, the scaffoldins contain one type of cohesin (type I or type II); although ScaB2 and ScaO1 carried both types of cohesin modules in their sequences. This phenomenon has been observed earlier in ScaDs of *A. cellulolyticus* and *C. clariflavum* [16,17,18].

The largest *C. alkalicellulosi* scaffoldin, ScaA, contains 10 type I cohesin modules, one CBM3, and one X-dockerin (XDoc) modular dyad at the C-terminus. The CBM3 is responsible for binding of the cellulosome and bacterial cell to the cellulosic substrate [40,46,47], while the XDoc has been shown to bind to type II cohesins [48,49]. With respect to modular arrangement, the organization of this polypeptide is similar to the primary ScaA scaffoldins of *C. thermocellum*, *A. cellulolyticus*, *C. clariflavum*, and *B. cellulosolvens* [17,18,22,45]; among which, ScaA also contained the highest number of cohesin modules of all scaffoldins in the different species.

In four *C. alkalicellulosi* scaffoldins (ScaB2, ScaD, ScaC, and ScaF1), three repeats of an SLH domain were detected, whereby the resultant SLH module would presumably exhibit an anchoring function of specific enzymatic units and/or primary scaffoldins to the bacterial cell surface via their resident cohesin(s). The latter cohesin modules were positioned in different branches of the phylogenetic tree (Figure 2), suggesting differences in both specificity and functionality. This could lead to a variety of enzymes/proteins and cellulosome architectures displayed on the cellular surface.

As opposed to the cell-associated scaffoldins, other scaffoldins could play a role in the assembly of cell-free cellulosomal systems. In this context, ScaB1 and ScaE contain three and four type II cohesin modules, respectively, in their polypeptide chains without additional modular components. This arrangement is similar to ScaE found in the cellulosomal systems of *C. thermocellum*, *A. cellulolyticus*, *C. clariflavum*, and *B. cellulosolvens*, all of which contained seven type II cohesin modules in their respective sequence [16,18,22]. In addition, ScaF2 comprises a single type II cohesin and a module of unknown function. ScaG contains one type I cohesin module and one module identified as a copper-amine-oxidase-like domain, which has been linked to a cell-surface anchoring function [16,18,50] and renamed “cell surface-binding module” (CSBM). An orthologous type of ScaG scaffoldin is found in the genomes of other cellulosome-producing bacteria, including *C. thermocellum*, *A. cellulolyticus*, *C. clariflavum*, and *B. cellulosolvens* [16,18,22,45].

ScaK and ScaN1-7 could be “adaptor scaffoldins” as ScaC in *R. flavefaciens* [14] and *Ruminococcus champanellensis* [51,52], as they contain one to three copies of type I cohesins and a C-terminal type I dockerin module in their sequences, except for ScaN7 that has a type I dockerin at its N-terminus.

*C. alkalicellulosi* scaffoldins O1, O2, P1, and P2 were similar to the ScaOs and ScaPs from *A. cellulolyticus* and *B. cellulosolvens*. They all contained at least one predicted peptidase, a dockerin, one or more cohesins, and several auxiliary module(s), which include a fibronectin type III domain (Fn3), modules with homology to Rhs repeat domains (Rhs), bacterial pre-peptidase C-terminal domains (PPC), von Willebrand factor type A domain (WFA), and enzymes, i.e., galactose oxidase [16]. ScaO1 had both type I and type II cohesin modules (reminiscent of the modular arrangements of *A. cellulolyticus* ScaO and ScaP), whereas ScaO2 contained only one type II cohesin module that shows close homology to that of *A. cellulolyticus* ScaO. The two ScaP cohesins appeared on the same branch of the phylogenetic tree together with ScaP of *A. cellulolyticus*. Unlike the others, ScaP2 contained a type II X-dockerin modular dyad in its polypeptide chain. The combination of these modules formed a unique feature and organization, which resembled the type of modular components and architecture observed in ScaO and ScaP of *A. cellulolyticus*.

### 3.4. Catalytic Subunits and Scaffoldins Contain Dockerin Modules, which Share Similar Features to those of C. thermocellum and A. cellulolyticus

**Figure 3:**
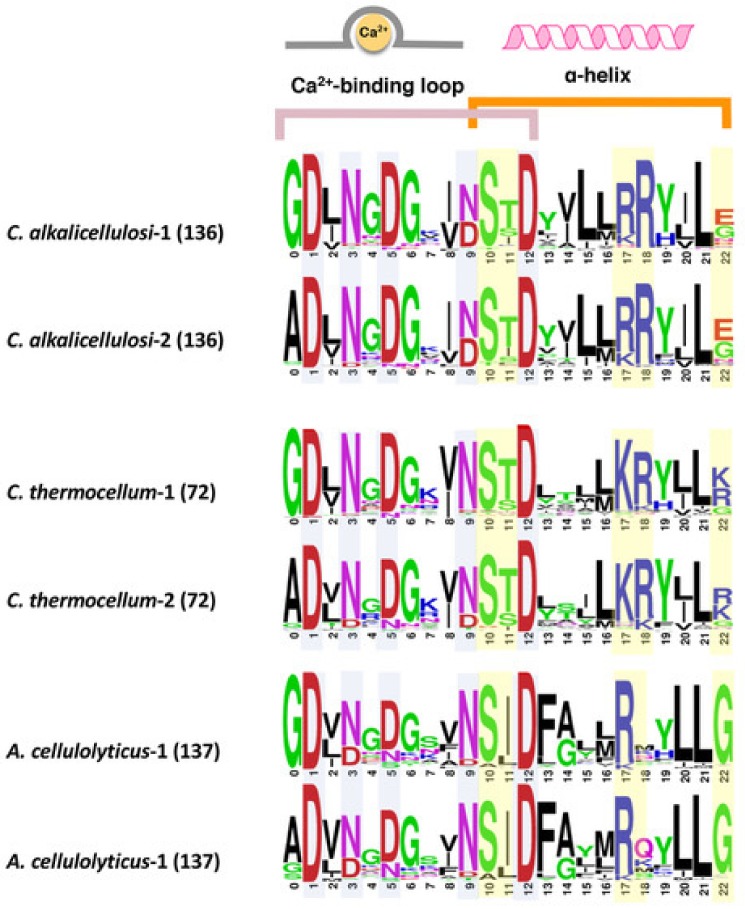
Comparison of conserved type I dockerin sequences of *C. alkalicellulosi*, *C. thermocellum*, and *A. cellulolyticus*. Each sequence shows the repeated segments (1 and 2) of dockerin sequences. Light blue and yellow colors highlight potential residues responsible for calcium binding and recognition sites for cohesin–dockerin interaction, respectively. The number in the bracket represents the numbers of sequences in the given species used for creating alignment in WebLogo.

The genome of *C. alkalicellulosi* encoded an abundance of dockerin-containing proteins. Of the 142 putative dockerin sequences, 138 were classified as type I and four as type II, based on the comparison of the *C. alkalicellulosi* dockerin sequences with those of *C. thermocellum* and *A. cellulolyticus* (Figure 3, Supplementary Table S2). Among the type I dockerin-containing proteins, 57 sequences were found to carry catalytic modules involved in polysaccharide degradation. This includes 57 enzymes from 15 different glycoside hydrolase (GH) families, five carbohydrate esterases (CEs), and one polysaccharide lyase (PL), based on CAZy classification [35] (Supplementary Table S2). Several dockerin-bearing predicted peptidases were also observed. Some sequences carried more than one catalytic module, indicating that they would display multifunctional enzymatic activities. In addition, the type I dockerin modules were widely dispersed among 11 of the scaffoldins (Figure 1). A few sequences exclusively contained non-catalytic modules, such as CBMs, serpins, and expansin-like modules (Supplementary Tables S2 and S4). The *C. alkalicellulosi* genome is the second (after *C. clariflavum*) shown to encode dockerin-associated expansins [53]. This reinforces the credibility of the contention that the presence of dockerin-bearing expansins is an authentic feature of some cellulosome-producing bacteria.

In general, the numbers of genes encoding for the different GH families in the *C. alkalicellulosi* genome were very similar to other complex cellulosome-producing species (Supplementary Table S4). Like the other clostridial species, the *C. alkalicellulosi* genome encoded for two GH48 cellulases (one dockerin-bearing and the other not) and large numbers of enzymes from GH5 and GH9 (mainly predicted cellulases). The lone striking exceptions, compared to the other complex cellulosome-producing species, were the exceptionally high numbers of GH families 10 and 11 in *C. alkalicellulosi*, which clearly indicate that the bacterium would exhibit a markedly elevated capacity to degrade xylanase substrates. This is further reflected in the conspicuously high numbers of CBMs from families 6 and 22 (Supplementary Table S4). In this bacterium, the CBM6s were largely associated with putative xylanases and other hemicellulases (i.e., GH families 8, 10, 11, 16, 30, and 43), whereas all of the *C. alkalicellulosi* CBM22s were associated with GH10 enzymes. Four of the CBM4s appeared in a single predicted cell-surface GH16 laminarinase/licheninase, and another four were each associated with GH9 cellulases. Of note also were the six CBM2s, which appeared in *C. alkalicellulosi* hemicellulases, as opposed to the scaffoldin-borne CBM2s of *A. cellulolyticus* and *C. clariflavum*. Interestingly, the *C. thermocellum* genome lacked CBM2s.

Dockerin sequences typically possess a unique conservation pattern. The domain contains two repeats of a predicted Ca^2+^-binding loop and an α-helix, whereby the two repeats are separated by a linker segment [42,54]. Despite the conserved characteristics, their internal sequences vary from species to species or even within the same species [51]. To reveal the sequence features of dockerins of *C. alkalicellulosi*, multiple sequence alignment of type I dockerin protein sequences was performed (Supplementary Table S2), and a sequence logo of the two repeats of the dockerin modules was created (Figure 3).

The highly conserved amino acid residues at positions 1, 3, 5, 9, and 12 are considered putative Ca^2+^-binding residues [54], whereas those at the positions 10, 11, 17, 18, and 22 are predicted recognition residues [55], which have been shown in other species to be significant sites for the tight binding interface between the cohesin and dockerin. Interestingly, the residues at positions 10, 11, 17, and 18 interact with the cohesin via the side chain, whereas the residue at position 22 interacts via the main chain [56]. Like *C. thermocellum* and *A. cellulolyticus*, it was observed that the two repeated segments in the type I *C. alkalicellulosi* dockerins were similar, which would indicate a dual- rather than single-mode of binding [57,58].

The major recognition residues of the *C. alkalicellulosi* and *C. thermocellum* dockerins were very similar, with S/T/R at positions 10/11/18, the major difference (R instead of K) being at position 17 (Figure 3). Position 22 was less conserved. In the case of *A. cellulolyticus*, the major recognition residues were also similar, but Ile replaced Thr in position 11, position 18 was largely variable whereas G was present at position 22. In several isolated cases, the major recognition residues were identical in dockerins of the three species.

### 3.5. Selection of Representative Cohesin/Dockerin Modules for Interaction Studies

Assessing cohesin–dockerin interactions is one means to investigate how *C. alkalicellulosi* would produce and assemble cellulosome complexes. In this respect, 21 cohesin and eight dockerin modules were selected as the representatives of *C. alkalicellulosi* cohesin/dockerin diversity and tested for binding interaction. The cohesin modules were chosen based on the divergent sequences, in order to examine the range of binding affinities. Dockerins were selected based on their origins or sequence particularities: (1) Dockerins carrying key cellulolytic enzymes, i.e., GH9 and GH48, in order to localize the catalytic subunits in the cellulosomes; (2) dockerins on scaffoldins, and (3) dockerins with unusual amino acids in the predicted recognition sites. Figure 1 shows the selected cohesin modules (labeled with a black dot).

To precisely detect interactions among the various cohesin and dockerin modules, the matching fusion protein system and affinity-based ELISA assay were employed [41]. This high-throughput method allowed us to examine the binding ability of an individual cohesin or dockerin towards a variety of its counterpart modules. By using this approach, the selected cohesins and dockerins were introduced into two different cassettes, in order to construct chimeric proteins. Each cohesin module was fused to CBM3a, derived from the CipA (ScaA) scaffoldin of *C. thermocellum* cellulosome, at its N-terminus forming CBM-Coh, while a dockerin module was integrated at the C-terminus of xylanase T6 from *Geobacillus stearothermophilus* with an additional N-terminal His-tag forming Xyn-Doc. These fusion domains not only offer the ease in purification through cellulose microbead-adsorption for CBM-Coh or a Ni-NTA affinity column for Xyn-Doc, but also increased the expression level in *E. coli* cells and dramatically improve solubility and stability of the proteins. This improvement was consistent with previous studies [17,18,51]. In addition, primary antibodies directed towards these fusion tags form the basis for the evaluation of the cohesin/dockerin interaction by ELISA.

### 3.6. Profiles of Cohesin–Dockerin Interactions

**Figure 4:**
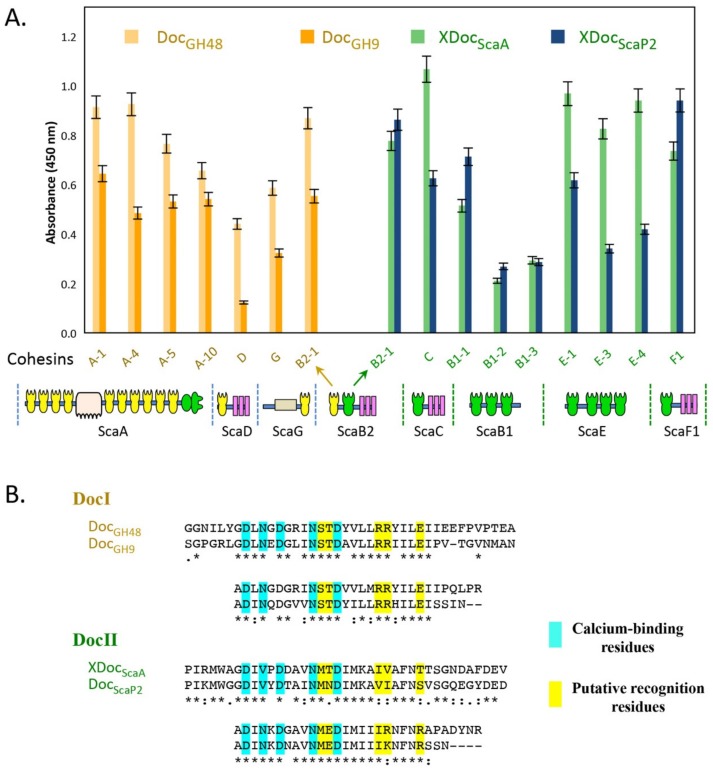
(**A**) Determination of cohesin–dockerin interaction based on ELISA assay. ELISA plates were coated with the designated XynDoc fusion proteins at a concentration of 1 μg/mL, and the indicated Coh constructs were examined at a reference concentration of 100 ng/mL. Cohesin A-1, A-4, and A5 indicate the first, fourth, and fifth cohesin in scaffoldin ScaA. The other cohesins in the figure are named similarly. See Figure 1 for description of the scaffoldins. (**B**) Type I and type II dockerin sequences used in this study.

In order to give an exploratory view of the cellulosome system of *C. alkalicellulosi*, a total of 168 cohesin and dockerin interaction tests were performed. The type I cohesins from the ScaA scaffoldin and the cell-surface anchoring scaffoldins, namely ScaB2, ScaD, and ScaG, were priority targets. On the one hand, the former was expected to be a core protein that accommodates the key cellulosomal enzymes, and the latter would attach significant enzymes onto the cell surfaces. By sequence analysis, these cohesins were expected to interact with type I dockerins; thus, the type I dockerins of GH48 (Xyn-Doc_GH48_) and GH9 (Xyn-Doc_GH9_) were chosen to test for the binding interaction (Figure 4).

Xyn-Doc_GH48_ bound significantly to all four selected cohesin modules of ScaA despite minor differences in their affinity. Cohesin modules 1 and 4 exhibited relatively higher interaction with Xyn-Doc_GH48_ than others, suggesting that they may be preferable sites for this enzyme to integrate into ScaA. Xyn-Doc_GH9_ also bound to all cohesins tested; however, their interaction intensities were comparatively lower than those of Xyn-Doc_GH48_ at the same positions. This may possibly reflect the difference in recognition residues between the two dockerin sequences. Both Xyn-Doc_GH48_ and Xyn-Doc_GH9_ interacted with ScaG, whereby Xyn-Doc_GH48_ exhibited relatively higher binding affinity. Notably, Xyn-Doc_GH9_ showed relatively poor binding affinity to ScaD.

The second cohesin module of ScaB2 and the cohesins of ScaB1, ScaC, ScaE, and ScaF1 were all bioinformatically identified as type II cohesins. ScaB2, ScaC, and ScaF1 may be anchored to the cell surfaces via their SLH modules. In contrast, ScaB1 and ScaE contained only cohesin modules, suggesting their free-states in the environments. However, since this is a draft genome, and the linker residues of the scaffoldins are notoriously difficult to sequence, the possibility remains that ScaB1 and ScaE may be fragments of a larger cell surface-anchoring scaffoldin, like in the other complex cellulosome-producing species.

The X-dockerin modular dyad (Xyn-XDoc_ScaA_), which carries the primary scaffoldin ScaA, bound strongly to the type II cohesin modules of ScaB2, ScaC, ScaE, and ScaF1 (Figure 4). In ScaB1, some differences in binding could be discerned among its cohesins, and their binding to the Xyn-XDoc_ScaA_ was relatively low. Like in other cellulosome-producing species, the bacterium likely provides two distinct cellulosome systems: One that attaches the ScaA to the bacterial cell walls, which is widely known as a cell-associated system and a cell-free system, released to the media.

The type II dockerin module of ScaP2 (Xyn-XDoc_ScaP2_) was also tested for interaction against the same type II cohesins. This scaffoldin is of interest because it not only carries a cohesin module, but also bears two peptidases, one galactose oxidase, one Rhs-repeated domain, and one unknown module. This makes ScaP2 unique and its modular organization indicates an unconventional type of scaffoldin, not yet reported.

All representative type II cohesins from the different scaffoldin interacted with Xyn-XDoc_ScaP2_. Among them, type II cohesins of ScaB2 and ScaF1 showed relatively high binding affinity, similar to that of the XDoc_ScaA_. The cohesins of the putative cell-free scaffoldins, ScaB1 and ScaE, exhibited comparatively lower levels of binding with Xyn-XDoc_ScaP2_, although some differences were observed that could possibly reflect their relative positions on the phylogenetic tree (Figure 2).

ScaN1 to 6 individually contained two or three type I cohesin modules and one type I dockerin module in their polypeptide. Phylogenetic analysis revealed that the type I cohesins of ScaN1 to 6 reside in the same cluster with ScaA and ScaG cohesins. Thus, these cohesin modules may give similar pattern of cohesin–dockerin interaction as were observed in ScaA and ScaG. On the other hand, by comparison, the type I dockerin sequences of ScaN1 to 6 shared some sequence similarity with the type I dockerins bearing enzymes, except for amino acids at the recognition sites. Thus, it is possible that these scaffoldins bearing type I dockerins bind to the cohesin modules in a similar manner to enzyme-borne type I dockerins, or exhibit different binding affinities.

The cohesin modules 1 of ScaN3 and ScaN4 were chosen as representatives to examine the binding ability towards the Xyn-Doc_GH48_ and Xyn-Doc_GH9_ (Supplementary Figure S1). It was found that both Xyn-Doc_GH48_ and Xyn-Doc_GH9_ bound to these two cohesin modules with high intensity, indicating their important role for integrating the enzymes into the scaffoldins.

Since *C. alkalicellulosi* inhabits alkaliphilic environments with a pH of around 10 [27], one might expect the difference in the optimal pH for the cohesin and dockerin interactions compared to those of neutrophiles (i.e., bacteria that thrive in neutral pH environments). In this respect, the suitable pH for cohesin–dockerin interaction was determined by varying the pHs of the buffer during the binding assay, ranging from 5.0 to 11.0. Four pairs of complimentary cohesins and dockerins were selected as representatives and tested for binding ability (Supplementary Figure S2). It was found that all of the cohesin–dockerin pairs showed similar profiles, by which the binding interaction increased from pH 4.0 to 7.0 (the optimum pH) and gradually decreased after pH 7.0. The relative intensities of the binding interactions all remained more than 60% at pH around 10, suggesting that the cohesins and dockerins were stable and remained functionally active under extreme pH conditions. Comparison between the binding activity between selected cohesins and dockerins from *C. alkalicellulosi* and those of two other cellulosome-producing bacteria (*A. cellulolyticus* and *B. cellulosolvens*) at high pH (pH 8 to 10; Supplementary Figure S3), indicated that all three interactions were relatively stable, but without any advantage of the *C. alkalicellulosi* system.

Since the *C. alkalicellulosi* dockerins shared some sequence similarities to *C. thermocellum* and *A. cellulolyticus*, we sought to examine possible cross-species interactions among the three species. In this study, type I dockerins of *C. thermocellum* and *A. cellulolyticus* and other microbial species were used as representatives, and their interactions were performed against type I cohesins (modules: 1, 4, 5, and 10) of the primary ScaA of *C. alkalicellulosi* (Supplementary Table S5). Compared to the interaction of Xyn-Doc_GH48_ against the same cohesin modules, the interactions of the *C. thermocellum* dockerin with the ScaA cohesins showed marginally lower intensity, while no interactions were observed for the dockerin from *A. cellulolyticus*. It is likely that the cross-reactivity might have occurred according to the high similarity in the sequence motif. The Ser/Thr motif at positions 10/11 was highly conserved in the type I dockerins of both *C. alkalicellulosi* and *C. thermocellum*, despite the relatively minor difference in the Arg/Arg motif at positions 17/18 (Lys-Arg motif for *C. thermocellum*). Intriguingly, the type I dockerin from *B. cellulosolvens* ScaA also interacted (albeit with relatively low activity) with the *C. alkalicellulosi* ScaA cohesins. Indeed, the putative recognition residues (positions 10/11, 17/18) of the *B. cellulosolvens* dockerin (Ser/Asp, Arg/Gln) [22] were similar to those of *C. alkalicellulosi*, which might well explain the observed results.

## 4. Discussion

Most of the cellulosome-producing bacteria isolated to date are neutrophilic, i.e., adapted to subsist and grow in environments where the pH is relatively neutral [24]. Here, we collected extensive information on the functionally active cellulosomal assemblies of the alkaliphilic, cellulolytic bacterium, *Clostridium alkalicellulosi*, isolated from an alkaline environment. Our in-depth investigations of both structural genes of the cellulosomal system and biochemical evidence of the cellulosome assembly, serve to establish that *C. alkalicellulosi* is a true cellulosome-producing bacterium, thereby providing proof of previously reported assumptions [28]. The bioinformatic and biochemical experiments performed here highlight the genomic potential of its cellulosomal architectures. Our genomic analysis of the draft genome indicated that the *C. alkalicellulosi* encoded for 22 cellulosomal cohesin-bearing scaffoldins and 142 dockerin-containing proteins.

Most of its scaffoldins share some similarity in terms of modular arrangements to the scaffoldins of *C. thermocellum* and *A. cellulolyticus*. Out of the 43 *C. alkalicellulosi* cohesins (including both types I and II), 35 of them (81%) were closely related to those of *C. thermocellum*, and eight cohesins (9%) were related to cohesins of *A. cellulolyticus* and *C. clariflavum*. This may in part infer a similar ancestor for *C. alkalicellulosi* and *C. thermocellum*, which is in accordance with the phylogenetic lineage based on 16S rRNA gene analysis, which shows the close relationship between the two species (95.5% identity to *C. thermocellum* and 94.8% identity to *A. cellulolyticus*) [27].

Some scaffoldins, however, exhibited an uncommon arrangement as opposed to the known (classical) scaffoldins. This includes ScaO1, ScaO2, ScaP1, and ScaP2. Besides the cohesin modules, these scaffoldins contain dockerins and additional domains, such as Fn3, Rhs, PPC, WFA, peptidases, galactose oxidases, and domains of unknown function. Such an unconventional scaffoldin was observed earlier only once in *A. cellulolyticus* ScaO [16]. Scaffoldins bearing carbohydrate-active enzymes in their polypeptide chains have been observed earlier, such as the GH9 in the primary ScaA of *A. cellulolyticus* [16], a GH44 in *B. cellulosolvens* ScaR3 [22], and GH25 lysozyme in *R. champanellensis* ScaK [51,52]. Nevertheless, galactose oxidases are rarely found in bacteria in general [59,60]. In *C. alkalicellulosi* scaffoldins, their presence could imply a saprophytic life style in the soda lake [26,27]. In this context, it has been reported that the degradation of the flora surrounding the soda lake produces a source of cellulose, associated with galactose-containing hemicelluloses [26,27,29,61,62]. Thus, the galactose oxidases within *C. alkalicellulosi* scaffoldins could catalyze the oxidation of galactose oligomers and polymers [59] and have a role in polysaccharide modification [63]. In addition, the production of H_2_O_2_ by the galactoside oxidases may be important for function in the soda lake environment [63,64]. However, the catalytic reaction of galactose oxidase on polysaccharides comprises oxidative and reductive half-reactions, requiring molecular oxygen as an electron acceptor [65], which contradicts the anaerobic lifestyle of *C. alkalicellulosi*. Thus, further research on expression and function of these genes would be required to understand their role as a cellulosome component in an anaerobic environment.

Given that (i) the microenvironments around the recognition sites between cohesin–dockerin interfaces are neutral and (ii) the protein surfaces are dominated by negatively charged amino acids, the acidic residues are thus deprotonated under high pH environments and have higher capacity to bind water molecules, making them hydrated [66]. This might explain the possible mechanisms of high pH adaptation of these specific proteins [66]. It is also of interest to investigate the mechanism of formation of cellulosome complexes in soda lakes where Ca^2+^ is limited [26], since it is well established that calcium is important for cellulosomal assembly, stability, and activity [67].

The natural association of peptidases and cellulases and additional carbohydrate-active enzymes found in scaffoldins ScaO1, ScaO2 ScaP1, and ScaP2 could serve in the removal of proteins embedded in the biomass substrate that prevent access to the lignocellulose. Annotations for peptidases were also reported in *A. cellulolyticus* and *B. cellulosolvens* scaffoldins, and some studies have demonstrated the presence of genes encoding dockerin-bearing peptidases in the bovine rumen metagenome [68], and in the genomes of *C. thermocellum* and *R. flavefaciens* (subtilisin-like serine protease [69] and cysteine peptidase [70], respectively). In addition to their potential role in processing biomass, the cellulosomal peptidases could play a key role in cellular nitrogen utilization and could complement the microbial activity and the *nifH* gene encoding a nitrogenase observed in *C. alkalicellulosi* [26,27]. Thus, *C. alkalicellulosi* and the cellulosome systems may in part contribute to nitrogen cycling in the soda lake. Indeed, nitrogen fixation generally occurs under anaerobic environments—conditions that favor growth of the bacterium [26].

**Figure 5:**
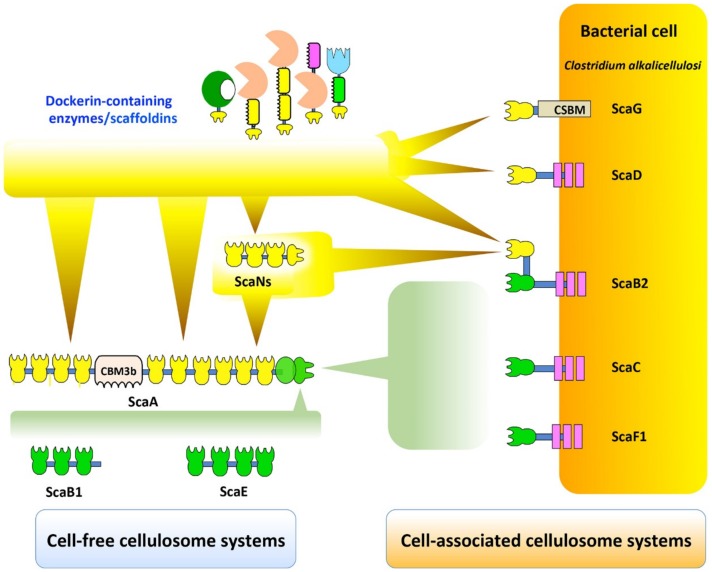
Exploratory view of cell-free and cell-associated cellulosomal systems in *C. alkalicellulosi*. The scheme represents the putative interactions among scaffoldins, cohesins, and dockerins, according to the specific binding study by affinity-based ELISA (Figure 4 and Figure S1). At least six types of complex formation occur. Type I dockerin-containing enzymes are able to bind type I cohesins of: The primary scaffold ScaA, ScaN, and cell-associated scaffoldins, namely, ScaG, ScaD, and ScaB2. Additionally, type I dockerin-borne ScaN is also able to bind type I cohesins of the primary ScaA, thus increasing number of enzymes on ScaA. ScaA binds cell-associated ScaB2, ScaC, and ScaF1, as well as cell-free ScaB1 and ScaE, through type II cohesin–dockerin interaction, resulting in cell-associated and cell-free cellulosome systems, respectively.

As presented in Figure 5, potential *C. alkalicellulosi* cellulosome architectures were revealed experimentally through the detected cohesin-dockerin interactions [41]. ScaB2, ScaC, and ScaF1 are localized on the bacterial cell surfaces by virtue of their SLH modules that interact with peptidoglycans [71], and thus account for anchoring the complex displayed on the cell surfaces. The primary ScaA contains 10 type I cohesins, and can thus accommodate up to 10 enzyme subunits. The C-terminal type II dockerin of the ScaA specifically binds to type II cohesins of ScaB2, ScaC, and ScaF1, similar to the cell-surface anchoring of the *C. thermocellum* cellulosome [72,73]. However, it currently seems unlikely that *C. alkalicellulosi* would form extensive polycellulosomes on its surface, owing to the apparent lack of multiple cohesin-containing anchoring scaffoldins. Since ScaB2 contains an additional type I cohesin which could bind to the ScaN dockerins the maximum theoretical number of the enzymes would be at least 30.

Like the other complex cellulosome-producing bacteria, the presence of a cell-surface ScaG equivalent may indicate a special role as a carrier protein that may serve as a molecular shuttle in cellulosome assembly [50,74]. Interestingly a recent study showed that *C. clariflavum* ScaG preferably binds the type I dockerin-bearing expansin, a small protein that disrupts noncovalent-bonding between cellulose microfibrils and matrix polymers [53]. This would presumably facilitate cellulosome-mediated hydrolysis by loosening the plant cell wall structure [53,75]. The two *C. alkalicellulosi* cellulosomal expansins may thus have a biological function as suggested earlier [53]. It is still a mystery, however, why cellulosomal expansins are restricted to these two clostridial species and not a more common element in other cellulosome-producing strains.

ScaB1 and ScaE contain three and four type II cohesin modules, respectively, but neither contains SLH modules nor CBMs. Structurally, they are similar to the cell-free heptavalent ScaE scaffoldins of *C. thermocellum*, *A. cellulolyticus*, *C. clariflavum*, and *B. cellulosolvens*, all of which contain seven type II cohesin modules and lack other modules. Theoretically, the interaction of ScaA with ScaB1 or ScaE would result in large polycellulosomal complexes, which would integrate up to 40 enzymatic subunits. Nevertheless, a lack of a complete genome, combined with possible assembly errors, lends a degree of uncertainty as to the status of these relatively small scaffoldins and whether they may be a part of a larger surface-anchoring scaffoldin (i.e., ScaC or ScaB2). This is always a danger when working with draft genomes, but the knowledge gained from such studies, albeit error prone, far exceeds such dangers, as long as the uncertainties are recognized and taken into account.

## 5. Conclusions

In this study, *C. alkalicellulosi* cellulosomal architectures were revealed in which the extensive scaffoldin repertoire provides a variety of sophisticated cellulosomal systems. The unconventional scaffoldins containing peptidases and oxidative enzymes appear to be associated with both cell-associated and cell-free systems. This raises the question as to their function(s) in the cellulosomal system, as well as the nature of the microorganism and its role in the alkaline soda lake ecosystem. Insights into the basic structure and properties of the cellulosome in this bacterium, however, extends our knowledge of the cellulosome paradigm from different microbial species and could lead to the development of alkali-stable designer cellulosomes [76,77] with potential applications in paper and pulp, detergent, and biofuel industries.

## Figures and Tables

**Table 1:** Putative cohesin-borne scaffolding protein of *C. alkalicellulosi*. The type I and type II cohesins (**Coh1** and **Coh2**) are shown in bold font in the table, since the cohesin module is the definitive scaffoldin component.

Protein ID	Designated Name	Modular Arrangement	Remark
CloalDRAFT_3068	ScaA	SIGN **Coh1 Coh1 Coh1 Coh1 CBM3 Coh1 Coh1 Coh1 Coh1 Coh1 Coh1** XDoc2	Sequence homology
CloalDRAFT_3067 CloalDRAFT_3066	ScaB1 ScaB2	SIGN **Coh2 Coh2 Coh2** **Coh1 Coh2** SLH	Sequence homology, clustered on the genome following the *scaA* gene.
CloalDRAFT_3065	ScaC	**Coh2** SLH	Clustered on the genome with the *scaA* gene.
CloalDRAFT_3064	ScaD	SIGN **Coh1** SLH	Single type I cohesin, clustered on the genome with the *scaA* gene.
CloalDRAFT_0628	ScaE	SIGN **Coh2 Coh2 Coh2 Coh2**	Similarity of Coh2 sequences with ScaE, lack of SLH
CloalDRAFT_3961	ScaF1	**Coh2** SLH	Type II cohesin, SLH
CloalDRAFT_0629	ScaF2	SIGN UNK **Coh2**	High sequence homology to Ac-ScaF
CloalDRAFT_4206	ScaG	SIGN CSBM **Coh1**	Sequence homology, CSBM
CloalDRAFT_0457	ScaK	SIGN Coh2 Doc1	Sequence homology
CloalDRAFT_2910	ScaO1	Peptidase PPC **Coh1** Doc1 **Coh2** RhsA	Modular similarity to *Ac*-ScaO
CloalDRAFT_1780	ScaO2	SIGN Peptidase **Coh2** Doc1 GalOx RhsA	Close sequence homology with *Ac*-ScaO cohesin
CloalDRAFT_3207	ScaP1	SIGN Doc1 **Coh2** Peptidase FN3 WFA	Sequence homology
CloalDRAFT_1967	ScaP2	SIGN Peptidase **Coh2** Peptidase XDoc2 GalOx RhsA UNK	Sequence homology, modular similarity to *Ac*-ScaP
CloalDRAFT_2305	ScaN1	SIGN **Coh1 Coh1 Coh1** Doc1	High sequence homology to *Ct*-ScaA, but containing type I dockerin
CloalDRAFT_0274	ScaN2	SIGN **Coh1 Coh1** Doc1	
CloalDRAFT_3567	ScaN3	SIGN **Coh1 Coh1** Doc1	
CloalDRAFT_3500	ScaN4	SIGN **Coh1 Coh1** Doc1	
CloalDRAFT_3290	ScaN5	**Coh1 Coh1** Doc1	
CloalDRAFT_2020	ScaN6	SIGN **Coh1 Coh1** Doc1	
CloalDRAFT_0656	ScaN7	SIGN Doc1 **Coh1**	

Abbreviations: *Ac*, *Acetivibrio cellulolyticus*, CBM3, family 3 CBM; Doc, type I dockerin; XDoc2, X-module paired with a type II dockerin; Coh1, type I cohesin; Coh2, type II cohesin; CSBM, cell surface-binding module; *Ct*, *C. thermocellum*; Fn3: Fibronectin type III domain; GalOx, galactose oxidase; peptidase, predicted peptidase; PPC, bacterial pre-peptidase C-terminal domain; Rhs, Rhs repeat domain; SIGN, signal peptide; SLH, S-layer homology module; UNK, X, module of unknown function; WFA: von Willebrand factor type A domain.

## Supplementary Materials

The following are available online https://www.mdpi.com/2076-2607/7/9/347/s1.

## Abbreviations

CBM, carbohydrate binding module; CSBM, cell surface-binding module; DNS, dinitrosalicylic acid; SLH, surface layer homology; Fn3, fibronectin type III domain; PPC, bacterial pre-peptidase C-terminal domain; Rhs, Rhs repeat domain; WFA, von Willebrand factor type A domain; XDoc, X-module coupled with type II dockerin; ORF, open reading frame; GH, glycoside hydrolase; Xyn, xylanase.
