# Supplementary material for: The Cellulosome Paradigm in An Extreme Alkaline Environment
Source: Microorganisms. 2019 Sep 12;7(9):347. doi: 10.3390/microorganisms7090347 (PMC6780208; doi:10.3390/microorganisms7090347)
Supplement: Supplementary file 1 [file microorganisms-07-00347-s001.zip › Suppl. Figures S1 S2 and S3.docx]

**
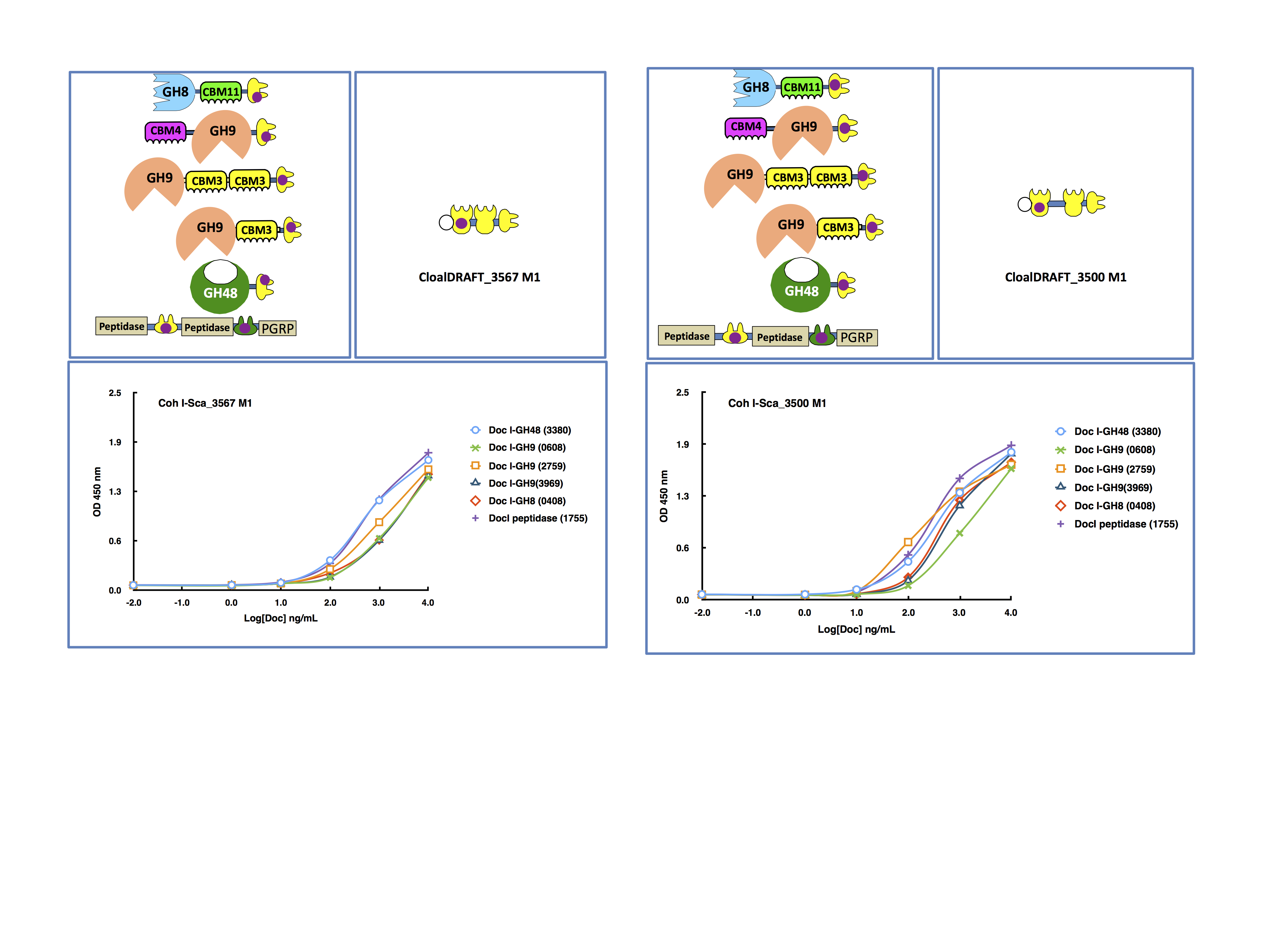
**

**Figure S1. ELISA experiments showing the interactions of cohesins 1 of ScaN3 (Sca3567) and ScaN4 (Sca3500) towards several dockerin-bearing enzymes.**

**
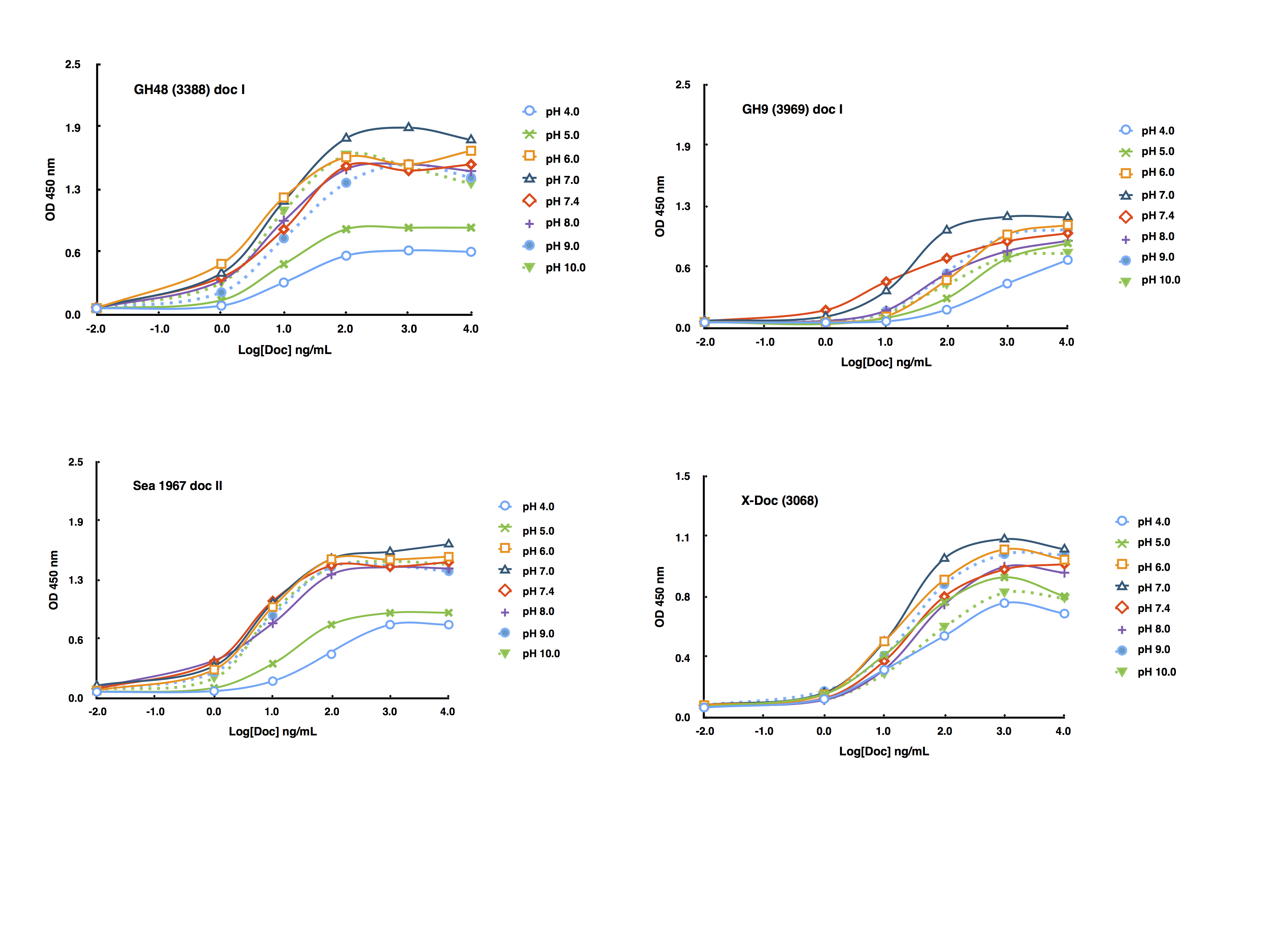
**

**Sca 1967 doc II**

**Figure S2. Effect of pH on cohesin and dockerin interactions.**

**Figure S3. pH dependence of Coh-Doc interactions.** Bc indicates the interaction between CohB and DocB of *B. cellulosolvens;* Ac for CohA and DocC3 of *A. cellulolyticus;* Ca for CohA and Doc_GH48_ of  *C*. *alkalicellulosi*. The binding interaction was performed by affinity-based ELISA as described in the Materials and Methods Section of the main text. ELISA pates were coated with Coh at a concentration of 1 μg/mL, and the Doc constructs were examined at a reference concentration of 100 ng/mL.
