# Supplementary material for: The Cellulosome Paradigm in An Extreme Alkaline Environment
Source: Microorganisms. 2019 Sep 12;7(9):347. doi: 10.3390/microorganisms7090347 (PMC6780208; doi:10.3390/microorganisms7090347)
Supplement: Supplementary file 1 [file microorganisms-07-00347-s001.zip › Table S1.docx]

**Additional File 1:**

**Table S1**: **List of primers used for amplification of *C. alkalocellulosi* cohesin and dockerin modules.** Restriction enzyme sites are capitalized and shown in bold.

| **Primer name** | **Nucleotide sequence** |
| --- | --- |
| *Cohesin module* | |
| ScaA-U-3068 coh1_BamH I  ScaA-L-3068 coh1_ Xho I | aattaa**GGATCC**tcacaagaactaacagtaac  ataatt**CTCGAG**ttgtgttgaacctaaattaa |
| ScaA-U-3068 coh4-CBM3_BamH I  ScaA-L-3068 coh4-CBM3_Xho I | aattaa**GGATCC**ggaagtggattaactgtaac  ataatt**CTCGAG**aggctccgtaccccaaacta |
| ScaA-U-3068 coh5_BamH I  ScaA-L-3068 coh5­_Xho I | aattaa**GGATCC**gcaaatgcattaagagttgg  ataatt**CTCGAG**tattgaacctccaacattta |
| ScaA-U-3068 coh10_BamH I  ScaA-L-3068 coh10­_Xho I | aattaa**GGATCC**gacggatttggagtaatagt  ataatt**CTCGAG**tactacagtacttccaacat |
| ScaB1-U-3067 cohII-1_BamH I  ScaB1-L-3067 cohII-1_ Xho I | aattaa**GGATCC**gatacaacttcaagtataga  ataatt**CTCGAG**tttaattggctcaggctgta |
| ScaB1-U-3067 cohII-2_BamH I  ScaB1-L-3067 cohII-2_ Xho I | aattaa**GGATCC**tcaggtgaaatagcaatgga  ataatt**CTCGAG**tttaattggctcaggctgta |
| ScaB1-U-3067 cohII-3_BamH I  ScaB1-L-3067 cohII-3_ Xho | aattaaGGATCCtcaggtgaaatagcaatgga  ataattCTCGAGtttaattggctcaggttgta |
| ScaB2-U-3066 cohI_BamH I  ScaB2-L-3066 cohI_ Xho I | aattaa**GGATCC**aatataacagttggattagt  ataatt**CTCGAG**aagcacaaaatttattgggt |
| ScaB2-U-3066 cohII_BamH I  ScaB2-L-3066 cohII_ Xho I | aattaa**GGATCC**gagcaaactccaatgccaga  ataatt**CTCGAG**tgctttaattggtaatggtt |
| ScaC-U-3065 coh2_BamH I  ScaC-L-3065 coh2_ Xho I | aattaa**GGATCC**tcaaaaatatacattgattt  ataatt**CTCGAG**ttctggttgtataactttat |
| ScaD-U-3064 Xho I  ScaD-L-3064 BamH I | aattaa**CTCGAG**agtaatatttttaaggttga  ataatt**GGATCC**gctaactcttacacttcctc |
| ScaE-U-0628 cohII-1_BamH I  ScaE-L-0628 cohII-1_ Xho I | aattaa**GGATCC**attgtgttagaagtagacaa  att**CTCGAG**ttatggttgtactactgagtaat |
| ScaE-U-0628 cohII-3_BamH I  ScaE-L-0628 cohII-3_ Xho I | aattaa**GGATCC**gatggatatatagaaatgac  att**CTCGAG**ttaaattgtttggggttgtataa |
| ScaE-U-0628 cohII-4_BamH I  ScaE-L-0628 cohII-4_ Xho I | aattaa**GGATCC**atatctatggtttttgacaa  att**CTCGAG**ttatagttcacctggctgtagta |
| ScaF1-U-3961 Xho I  ScaF1-U-3961 BamH I | aattaa**CTCGAG**gacaggacaacagcacaaaa  ataatt**GGATCC**tattacgttaccatgccaat |
| ScaG-U-4206 BamH I  ScaG-L-4206 Xho I | aattaa**GGATCC**gcagctaatcagtgggtagc  ataatt**CTCGAG**ttttgttatttttactcctc |
| ScaN1-U-2305 CohI-3_BamH I  ScaN1-L-2305 CohI-3_ Xho I | aattaa**GGATCC**gagatagagtatccaataat  att**CTCGAG**ttataatatttttaagtcattgt |
| ScaN3-U-3567 CohI-1_BamH I  ScaN3-L-3567 CohI-1_ Xho I | aattaa**GGATCC**gcattaaaagatttttctga  att**CTCGAG**ttatacttcatcagtaccagatt |
| ScaN4-U-3500 CohI-1_BamH I  ScaN4-L-3500 CohI-1_ Xho I | aattaa**GGATCC**gaagaagaaaacgagacttt  att**CTCGAG**ttattcatctgcaacaaatttgt |
| ScaN5-U-3290 CohI-1_BamH I  ScaN5-L-3290 CohI-1_ Xho I  ScaN5-U-3290 CohI-2_BamH I  ScaN5- L-3290 CohI-2_ Xho I | aattaa**GGATCC**tctgattttgatgcgggcga  att**CTCGAG**ttagggtgcagattctattatat  aattaa**GGATCC**aatactgtgtttcttcaaaa  att**CTCGAG**ttatagaatatgatcgaatactt |
| *Dockerin* | |
| ScaA-U-Cal3068-X-Doc KpnI  ScaA-L-Cal3068-X-Doc BamHI | aattaa**GGTACC**agaggatggttatactgtat ataatt**GGATCC**tctgttataatctgctggag |
| ScaK-U-0457-Doc I KpnI  ScaK-L-0457-Doc I BamHI | aattaa**GGTACC**acttgtgaaaatatataaat  ataatt**GGATCC**ttatgctggaaaagtctcag |
| ScaN1-U-2305-Doc I KpnI  ScaN1-L-2305-Doc I BamHI | aattaa**GGTACC**aaacaatgacttaaaaatat  ataatt**GGATCC**ttaattagctgggaaatcac |
| ScaN2-U-0274-Doc I KpnI  ScaN2-L-0274-Doc I BamHI | aattaa**GGTACC**aggagtgcatatagaaaaag  ataatt**GGATCC**ttatatattaagtattattc |
| ScaN7-U-0656-Doc II KpnI  ScaN7-L-0656-Doc II BamHI | aattaa**GGTACC**aaatgaagatttaagaccat  ataatt**GGATCC**ttaaacaggaaaactatcaa |
| ScaP2-U-1967-Doc II KpnI  ScaP2- L-1967-Doc II-1 BamHI | aattaa**GGTACC**acccataaaaatgtggggag  ataatt**GGATCC**ttaatttgaacttctgttaa |
| U-GH48_Cal3388 doc_KpnI  L-GH48_Cal3388 doc _BamHI | aattaa**GGTACC**atcaggaccaggcagattag  attaaa**GGATCC**ttagtttattgaagatattt |
| U-GH9_Cal2759-Doc I KpnI  L-GH9_Cal2759-Doc I BamHI | aattaa**GGTACC**aggtggtaatatattatacg  ataatt**GGATCC**ttaacgcggcagttgtggaa |
| U-GH9_Cal3969-Doc I KpnI  L-GH9-Cal3969-Doc I BamHI | aattaa**GGTACC**accaaatgattttatacttg  ataatt**GGATCC**ttagaagcttgttattattt |
| U-GH8-Cal0408-Doc I KpnI  L-GH8_Cal0408-Doc I BamHI | aattaa**GGTACC**accaaacatactatatggag  ataatt**GGATCC**ttaaccctgtgctggaaatc |
