# Supplementary material for: The Cellulosome Paradigm in An Extreme Alkaline Environment
Source: Microorganisms. 2019 Sep 12;7(9):347. doi: 10.3390/microorganisms7090347 (PMC6780208; doi:10.3390/microorganisms7090347)
Supplement: Supplementary file 1 [file microorganisms-07-00347-s001.zip › Table S2 .docx]

**Table S2.** Multiple sequence alignment of dockerins and protein domains of dockerin-containing enzymes/proteins.

| **ID number** | **Protein sequence** | **CAZymes and annotated protein domains** |
| --- | --- | --- |
| CloalDRAFT_0086  CloalDRAFT_0125  CloalDRAFT_0274  CloalDRAFT_0355  CloalDRAFT_0360  CloalDRAFT_0408  CloalDRAFT_0482  CloalDRAFT_0501  CloalDRAFT_0604  CloalDRAFT_0608  CloalDRAFT_0609  CloalDRAFT_0612  CloalDRAFT_0613  CloalDRAFT_0619  CloalDRAFT_0620  CloalDRAFT_0622  CloalDRAFT_0627  CloalDRAFT_0634  CloalDRAFT_0753  CloalDRAFT_0776  CloalDRAFT_0828  CloalDRAFT_0862  CloalDRAFT_0976  CloalDRAFT_0978  CloalDRAFT_1307  CloalDRAFT_1325  CloalDRAFT_1339  CloalDRAFT_1430  CloalDRAFT_1438  CloalDRAFT_1709  CloalDRAFT_1761  CloalDRAFT_1829  CloalDRAFT_1844  CloalDRAFT_1845  CloalDRAFT_1889  CloalDRAFT_1890  CloalDRAFT_1893  CloalDRAFT_2021  CloalDRAFT_2024  CloalDRAFT_2089  CloalDRAFT_2144  CloalDRAFT_2145  CloalDRAFT_2150  CloalDRAFT_2159  CloalDRAFT_2211  CloalDRAFT_2305  CloalDRAFT_2338  CloalDRAFT_2560  CloalDRAFT_2634  CloalDRAFT_2642  CloalDRAFT_2759  CloalDRAFT_2761  CloalDRAFT_2794  CloalDRAFT_3018  CloalDRAFT_3070  CloalDRAFT_3085  CloalDRAFT_3220  CloalDRAFT_3280  CloalDRAFT_3316  CloalDRAFT_3344  CloalDRAFT_3388  CloalDRAFT_3479  CloalDRAFT_3500  CloalDRAFT_3567  CloalDRAFT_3619  CloalDRAFT_3623  CloalDRAFT_3727  CloalDRAFT_3728  CloalDRAFT_3810  CloalDRAFT_3876  CloalDRAFT_3879  CloalDRAFT_3969  CloalDRAFT_3970  CloalDRAFT_4185  CloalDRAFT_4191  CloalDRAFT_0437  CloalDRAFT_0666  CloalDRAFT_0485  CloalDRAFT_0044  CloalDRAFT_0656  CloalDRAFT_1250  CloalDRAFT_1274  CloalDRAFT_1290  CloalDRAFT_1291  CloalDRAFT_1577  CloalDRAFT_1733  CloalDRAFT_1755  CloalDRAFT_0830  CloalDRAFT_0842  CloalDRAFT_0045  CloalDRAFT_4193  CloalDRAFT_3286  CloalDRAFT_3331  CloalDRAFT_2329  CloalDRAFT_2346  CloalDRAFT_2421  CloalDRAFT_2136  CloalDRAFT_2424  CloalDRAFT_2961  CloalDRAFT_3001  CloalDRAFT_3168  CloalDRAFT_3207  CloalDRAFT_3571  CloalDRAFT_3481  CloalDRAFT_3735  CloalDRAFT_4275  CloalDRAFT_4310  CloalDRAFT_4121  CloalDRAFT_4330  CloalDRAFT_4454  CloalDRAFT_4458  CloalDRAFT_1106  CloalDRAFT_1108  CloalDRAFT_1751  CloalDRAFT_0885  CloalDRAFT_0892  CloalDRAFT_2897  CloalDRAFT_2910  CloalDRAFT_3421  CloalDRAFT_1750  CloalDRAFT_3167  CloalDRAFT_2239  CloalDRAFT_1780  CloalDRAFT_4377  CloalDRAFT_4449  CloalDRAFT_0176  CloalDRAFT_4143  CloalDRAFT_3444  CloalDRAFT_4076  CloalDRAFT_3490  CloalDRAFT_0906  CloalDRAFT_1117  CloalDRAFT_0614  CloalDRAFT_0593  CloalDRAFT_0457  CloalDRAFT_3068  CloalDRAFT_1967  CloalDRAFT_1755 | **Type I Dockerins**  GDINGDNVVDSTDYILLRRYILEIINDFPQPN-------GRLAADVNNDGKIDSTDAILMRRYIMEIIDRFPA  GDVNLDGRVNSTDYVLIKRHILEMEGYKLECQ-------SFLAADIDRNGIIDTIDLVYMRRYILGIITTFPV  -DLNGDGVVNSTDSVLMRRYLLGIIDEFPVNN-------KMAVADLNGDGKINSNDYVLIKRIILNI------  GDLNYDDQIDSTDYILLKRYILGIAIFNTRDEENR----FKTSADLNGDGQVDSTDAVLLRRYILNIIDKLPV  GDINMDGKIDSIDYLMLRRHILVINTLTGD---------QFINADVNSDGKINSIDAVLLKRYILEIIDSF--  GDINGDGRIDSTDYALIRRHILEITVMSKGSE-------SYRRADVDGNGVVDSTDVILLRRYLLEIIDRFPA  GDINGDGKVDSTDYILLRRIVLEIPVANA----------NLEAADVNLDGAVNTTDCILLRRYLLEIIDSLPF  GDLNGDGVVDSTDLVILRRYILDIINSFPHPM-------AMISADVNGDGNINSTDYILIKRYILGIIDIFPV  GDLNKDQSVTSKDYSILKKYILEMTNDIPVHN-------KLDVADFNLDGKIDITDLIILKRYLLGLIKELPL  GDLNGDGVVNSTDCVLLRRYLLEIITEFPDKN-------GLKAADVDGNGTINSIDYVYMRRYVLSIISEFPV  GDLDGNGRIDSTDLVIMRRHILEIMYIKPE---------YLERADVNGDKVINSTDYILLKRYILGIITSFPA  GDLNGDGLIDSSDYILLRRYILGVTDSLQ----------NRAAADLNGDGLIDSIDVVLLRRYILEFISEFPA  GDLNGDGKIDSTDVILLRRHLLDITPLTET---------ALLNADLNKDGRIDSTDYALIRRYILGIISSF--  GDLNGDGRIDSTDIVLMRRYILEIIDGFSV---------PKEVADVNGDGVIDSSDYILMRRYLLEIITDFPV  GDLNGDGRVDSIDIVLLRRYILEIINGFSV---------PKEAADLNGDGVIDSTDYIILRRYLLEIISSFPV  GDLNGDGKIDSIDIVLMRRFILEIIDGFSV---------PLEAADVNGDGLINSSDYILMRRFLLEIITDFPS  GDLNNDGVINSTDYAKMIKHILRIERITDAN--------IHKAGDLNGDGAINSIDLVLLRRYILEIIDIFPV  GDVNGDGKIDSRDYVLLNRYILGIIDQFPCNN-------GITSADVDRNGQIDSTDYILLKRYLIEIIENLPV  GDLNGDELIDSTDVILLRRYIFEILNFTDDRERKL----FVTAADINGDGEVDSTDYILLKRFILEIPVNYPI  GDLNGDGLINSSDAVLLRRHILEIIELDSN---------AKKAADLNGDGVIDSTDFILLRRYILEIIPSLPV  GDLNNDGQIDSTDIVIMRRYILEILNDIP----------NIDAADLNGDGEIDSIDYTLLKRYILGIINKFPV  GDINGDGAINSTDYTLLRRVALGIVPVTDD---------ILAAGDLNKDGVINSTDLVLMRRYLLEIITSFN-  GDINNDGKIDSTDYILMRRHVLEITTLTGE---------SLLAADLNGDGKIDSTDCILLRRYILDIISIFPA  GDVDGDGRINSTDYALVRRFVLEIIDEFPSEY-------GHLAADVNGDGKIDSLDLILLRRYLLEIIDSF--  GDLDGNGSIDSTDYVLLRRHLLEISPLTGD---------ALLAADVNGDGVLDSTDYVILRRYLLDIISVFPA  GDLNGDGKVNSIDCVLLKRHLLELEGYTLEGE-------KLKAADLDANGSIDSTDYTLLKRFVLGIIDEFPA  GDVNGDNSVNSTDVNLMRRFILGIIDDFPYAE-------GKLAADVNGDGLINSTDYTLLRRYVLGIISTF--  GDLNGDGKVNSVDYVLLRRYLLEITTDI-----------NLLAADLNGDNLIDSTDYTILRRYLLEIIPSLP-  GDLDFNGSINSIDYVLAKRGVLQLGFPSDA---------AKLAADVNQDGQVNSTDIALLARYILGIIDRFPV  GDLNGDGVVDSIDYVYLKRYILGIGNTVYTDD-------WKRAADLNMDGSINSLDCVILKRFLLEIISEIPI  GDLNGDGKIDSTDYALLRRYILGIITSFPSEN-------GLISADVSGDGVIDSTDIVLMRRYLIEIIDKFPA  GDLNGDGKINSTDFVLLRRYLLGIIDEFSA---------SEKAADLNGDGKINSTDLVLLRRYLLVIIDRFPV  GDLNGDGIVNSTDYILLRRLVLEIPVNNV----------NLLAADVNADGLINSTDCIILRRYILEMIDTLPF  GDLNGDGKINSIDYVLLRRYLLEIIDSFPV---------AIEAADLNGDGKVNSTDLVILRRYLLVMIDRFPV  GDINNDGNIDSLDLILLRRYILSIIDDFPNAE-------ARYAADINGDGFINSTDYTLLRRYVLEIISEFPV  GDINGDNRIDSTDYVLLRRYVLGISSIPVED--------IYAVADLNLDGKIDSTDCIILRRYLLDIITSLPH  GDLNGDNRIDSTDYVLLRRYVLGISNIPSED--------KYAVADLNLDGRIDSTDCVILRRYLLEIIDSLPY  GDINGDGAVNSLDYVALRRHILEITILSGR---------SLQAADINGDGLVNSLDYVLLRRIILEII-----  GDINLDGKIDTTDLVLMRRYILEIIDKLPYEDMDNL---RIPIADVNGDGYINSTDYMLMRRYILEIITEFPV  GDINGDGLINSTDYILLRRYILEVTPSLPTTDVSGNPYRGDLAADLNGDGLIDSIDVILMRRYILEIITVFPV  GDLNNDGVIDSTDIILLRRYLLEIIDEFPTPY-------GEIVADVNRDGLINSTDYILLRRYILEIITSF--  GDLNGDGNIDSIDYMLLRRYILEIIDTLP----------NMDAADLNGDGKVDSTDAVLLRRYILEVISEFPR  GDLNGDGLINSTDVILLRRYILEILNFSNASERDL----FVTAADINGDGAVNSSDYILLRRFILEIPVSYPI  GDINGDGRVDSTDYILLRRIILEIPVSNA----------NLKAADVNLDGAVNTTDCILLRRYLLEIIDSLPF  GDINGDGEINSIDLVLLRRYILGVISSFDYEY-------GLEAADVNGDGNIDSLDYTIFRRYLLEIITEFKV  -DLNGDGVINSKDYVLLKRYILEIIDELPVN--------DISVADLNGDGKIDSVDYTLLRRYILEIISDFPA  GDVNGDGFINSIDYVLVKRYILGIIDKFPSKY-------GTLAADVDKNGSINSTDCVLIKRYILGIISEFEL  ADLNGDRKIDSTDAALMRRYILEIPVNFA----------NLEAADLNQDGVINSIDYVLLKRYILEIIW----  GDIDGNGKVDSSDYVLLRRYLLEIITSFNSPD-------GKKAADLDGNGEINSTDAILLRRYLLGIINKFPV  GDINGDGQIDSKDSAILRRILLDI---------------PVDGPVYIPEGIPLHELSRLIRNHILGISNF---  GDLNGDGRINSTDYVLLRRYILEIIEEFPV---------PTEAADLNGDGRINSTDVVLMRRYILEIIPQLPR  GDLNNDGKIDSSDYILLRRYLLNIIDEFPVD--------DIMAADLNGDGLINSTDAVLMRRYILGIINEF--  GDVNGDGKVDSSDYILIRRHVLNISSITDPI--------AFKAADVNGDGNINTLDVVLMRRYILEIIDMFPV  GDLNQDNVVNSLDLVLMRRYILNIITELSYD--------SKKAADINGDGKIDSTDYVILKRYILGIIDRL--  GDVNGDGIVNSIDYVLLRRFLLEIISDFPCPN-------GKLAADVNDDGVINSLDIVLLRRFLLEIISQF--  GDVTGNGIVGSKDYVLIRDYVSGKISEFPSPY-------GLLAADVDGDGEITINDVILVREMIQDRINKFPV  GDLNGDNLVNSTDCVLLRRYLLEIISEFPCEN-------GLMAADMNRDGKINSIDYVLLRRHVLGI------  GDLNGDKKINSADLVLLRRYILEIIDDIPA---------GLETADLNGDGKIDSLDYFTLKRFVLGIIKEFPV  GDLNGDGIVNSIDYVLMRRYILEIVSSLPVKD-------ELVAADLNLDGKINSNDYVILKRYLLEQIQKLPH  GDANYDGVIDSSDYVLLKRHILELSNMS-----------NKIICDLNYDGLINSTDLAILKRLILNIKTQ---  GDLNEDGLINSTDAVLLRRIILEIPVTGV----------NMANADINQDGVVNSTDYILLRRHILEISSI---  GDINGDGVVSSTDYILLKKHLNGKDGYILKGE-------KLLAADFNNTGSVNSSDLTSLKRYLLRSK-----  GDLNGDGVINSTDYVLLRRYVLEIIDDIPVGN-------VYHVADLNNDGKIDSTDCVILRKYILKIISSF--  GDLNDDGVINSTDYILLRRYILGINNDIPLEN-------KVDVADINGDGKIDSTDCVLLRKYILEITNL---  GDLNGDGNVDSTDFALLRRHILDITPLTGE---------NLLNADLNKDGKVNSTDYTLMRRYILGIISSFN-  GDINGDGSIDSTDVALLRRHLLQISGRILEGE-------ALLAADVNKDGSVNSTDYALMRRYILGIITEFPG  GDLNGDGNIDSTDVALLRRHLLNITPLSET---------ARSNADINKDGRIDSTDYVLMRRYILGIITSFPN  GDLNGDGKIDSTDVALLRRHLLGITTLTQA---------ALTNADINKDGRIDSTDYVLMRRYILGIITSF--  GDVSGDGNIDSTDCILLRRYLLGIIDSLPAQS-------WRVIADVNQDGKIDSTDYILMRRHVLGIIDLTTL  GDINGDGEINSIDLVLLRRYILGVISSFDYEY-------GLEAADVNGDGNIDSLDYTIFRRYLLEIITEFTV  GDLNGDDRIDSTDIVLLRRYILGIINEFPYPE-------GINAADVNADGVIDSSDYILLRRYLLGLITEFP-  GDLNGDGLVNSTDAILMRRHILEISVDI-----------NLQAADVNRDGVVNSTDYILLRRYILEIITSF--  GDLNQDGSVNSIDSVLLRRYILEIPVDI-----------DLESADLNGDGKIDSTDYILLRKFILEIIPSF--  GDINGDGVIDSTDYILIRRYLLGIITDFSYQH-------GRQAADINNDGQINSTDVALLRRYILGIIHSF--  GDLNGDGAIDSSDYILLRRYVLGVTTSLP----------NASAADLNGDGAIDSTDVILLRRYILELISAFPV  GDINDDGNVNSTDCVLLRRYMLEIIGKDRI---------NHKAADLNGDGNINSIDYVLLRRYVLGIIDIFPV  GDLNYDGRVSSTDYVIANRYILNGIDLKTTDVK------SFLASDVNGDGVFNSTDYVLIRRYILQLITSFPV  -DVNNDGVVNSTDAAIMKRYLLGIIDTFPVED-------EMLTADTNGDGVIDSTDYVLLRRYILGIIDKFPK  GDLNGDGRIDSTDIILMNRHVLGVSTLS-----------NTTVADLNGDGQINSTDYVLLRRYILEIISVFPV  GDLNGDGKINSIDLVLLRRYVLEIIDDFPVGN-------PFVYADLNGDGKVDSSDYILLRRYILNQIDSFPV  GDIFYDEEITSNDYVLLKRYILGIENTFPYAKGY-----ELLVADLNGDGKINSIDLVLLKRYILGLIDIFPV  GDVNGDGRFNSSDIILVRRYLLEIIKEFPSPN-------GLKAADVNGDGVINSIDYVLLRRRILEIINVFPV  GDLNGDGVVNSTDITLARRYLLEIISDFPSPN-------GLKAADVNGDGQINSTDYALLRRYILEIIDIFPA  GDLTGDGQINSSDVILLKRYLLSIIPNLPSQQ-------SIYAADLNADGKIDSTDYALLRRYVLGIMDVFPA  GDLNSDGNVNSTDAVLMRRFILGVINDDDI---------NTTNGDINGDGKIDSTDYVLLRRYVLEVINDLSK  GDVDGNGKIDSTDYVIMRRYVLGVIDSFQYEY-------AERAADVNGDNMIDSTDCVLMRRYLLGIITEFPV  GDINGDGLVNSTDYILLRRYILGISGVLDDAKKE-----FYYVADLNGDGLVNSTDCVLLRRYLLEIISKFPA  GDINNDGKIDSTDRVLLRRYMLDIIDESAI---------NIQAADLNGDGNIDSIDYVLLNRYLLEIIDVFPV  -DIDGNGYINSTDLVYMRRYLLEIIKELPVED-------KLWVADLNGDNLIDSIDYILLRRRVLNIISVFPK  GDLNGDGKINTIDLVMMNRHILEIQALE-----------DTVVADLNGDGKVNSIDYVLLRRYILEIISVFPV  GDLNGDGKVDSTDYILLRRYILNIIDEFPVG--------TLKVADLNGDDKINSTDAILMRRYILGIIDEFPV  GDLDGDGQVTSNDYQLLKRHVLGMKNITDSM--------QLKAADVNGDGKINSIDLVLIRRYILEVISAFPV  GDLNGDGRVNSIDLTILRRYLLEIIDINDIPISN-----FSIVADLNGDGRVNTTDYTLLKRYLLGIIDIFPV  GDLNNDGRIDSTDSVIMNRHILDIRAMD-----------DVSKADLNGDGMVNSTDYVLMKRYILGIISKFPV  GDINGDNKVNSTDLTLLRRYILEIISDFEVED-------NLWSADLNGDNLINSTDYALLKRYIIESITEFPK  GDLNDSGTVDSSDSVIMRRHLLDITQLTGE---------NIRRADLNGDGLIDSTDYVLLRRFILGIITEFPV  GDVNGDGRFNSTDIILVRRYLLEIIKEFPSPS-------GFKAADVNGDGLINSIDYVLLRRRILEIIKFFPV  GDINYDGKINSTDYILLKRYILNIDIPYLNGD-------ELLVADLNGNGKVNSIDLVLLRRYILGIIDVFPV  GDLNGDGNVDSTDYILIRRYILNIISDFPHPN-------GIIAADVNGDGLINSTDSILMRRFLLNVIDIFPV  GDLNGDGKINSIDLVLLRRYILDINSDFAVKD-------KLWSADVDGDNVIDSTDYVLIKRYILRVINEFPK  GDINHDGEINSTDLVLLRRHILGISEIKDSY--------GKIAADINGDDVLDSIDYILMRRAVLGMIR----  GDINGDGSINSTDYILLRRYILNIITEFPTSH-------GHIAADLNGDGNINSTDVILMRRYILNIIDEFPA  GDVNSDGSIDSTDCVLLRRYLLMISGIDSI---------NVKASDLNGDGSIDSTDYVLLRRYILRIIDEFPV  GDLNQDGVVNSTDYILLRRYVLGVIDEFPSEI-------AEIAADLNGDGVINSTDCVLLRRYILNMIDRFPV  GDLNGDGHVNSADYILLKRYVLGIITEFTSEH-------GEIVADLNGDGVINSTDCVLLRRYILEMIDKFPV  GDLNGDGIIDSTDYILLKRYVLSIITDFSPE--------QKIIADLNGDGVVNSIDCVLLKRYILDIINKFPV  GDLNNDGKVDSTDYILMRRYLLEIISDLPVPD-------KIWSADLNDDGKIDSTDAILMRRFLLEIISEFPK  GDLNGDGTVNSLDYILLQRYVLEIISEFPIKD-------GFVAADLNGDGKINSTDIVLMKRYILEIIDRFPA  GDLNYDGVINSTDYVLARRTFLTVDNLSVEDPK------LFKIVDLNGDGDFNTIDLVLMRRYILEIIDEFPG  GDLNGDGRVNSTDYVLIRRYILRIMENPSDE--------RMIAADLNGDGRLDSTDAVILRRMLLEILKYCPA  GDLNGDGRVNSTDYVLIRRYILRIMENPSDE--------RMIAADLNGDGRLDSTDAVILRRMLLEILKYCPA  GDLNGDGLINSTDIVLLRRYLLEIIKDFPSEN-------GKVAADLNGDGLINSTDIVLLRRYILNITKNFMA  GDLNGDGLINSTDIVLLRRYLLEIIKDFPSEN-------GKVAADLNGDGLINSTDIVLLRRYILNITKNFMA  GDLNGDGVIDSSDYILLRRFILGIISSIN----------NITAADLNGDGNIDSTDVILLRRYIMEIIRVFPV  GDLNGDGRVNSTDYVLIRRYILRIMENPSDE--------RMIAADLNGDGRLDSTDAVILRRMLLEILKYCPA  GDLNGDGIVNSIDAVLMRRYILGIATNIDI---------NTENADINGDGKINSIDYVLLKRYILEIINDLSV  GDINGDGEINSTDKVILRRYILGIISQLPNEY-------PLLVADLNGDGVINSSDYILLNRYILGVITEFID  GDINADGNINTSDYALLRRHILEISLLNTD---------QLSRADLNGDGNVNSTDLILLRRFILEIINVFPA  GDLNGDGRIDSSDVILLRRYILEIIDNFPV---------SKTAADLDGNGTINSTDYILLRRYVLEIIDVFPV  GDLNGDGKVDSSDYVLLRRHILGVTSLPGS---------VLSNADVNSDGNIDSTDVILMRRYILGIITTFPG  GDLNGDGKINSIDLVLLRRYILGIIDILPVED-------ELHSADLNNDGVINSSDYIILRRYILNIINELPF  GDINGDGVIDSKDYILLRRYILQVRTSLP----------NRIAADLNGDGNIDSIDVILLRRYILGIINKNPN  GDVNGDGVFNSSDRVLMLRYINEIIDTFPVED-------DYWAGDVTADGLINTSDYSMMGRVLMEIEDNFPK  YDVNGDGDVNSTDVVILTRYILEITTEL-----------PGEVGDFNGDGKIDSTDLVLLKRFILDGSYRFQ-  GDINGDGAIDSSDYILLRRYILQVATSLQ----------NRAAADLNGDGNIDSSDVILLRRYILQIIDRFPV  GDLNGDGVINSTDYILLRRYVLQVTTSLP----------NIDAADLNGDGRIDSSDVILLRRYILEIIDRFPA  GDLNGDGLINSTDVILLRRYVLEIISEFPAGGD------PLDVADLNGDGLINSTDVILLRRYVLEIIDKFPN  GDLNGDGKVDSSDYVLLRRHILGVTSLPGA---------VLSNADVNSDGNIDSTDVILMRRYILGIITTFPG  GDVNGDGRINSIDYILVKRYILGIITEFATED-------GLKAADVDNNGSINSIDLSYIRRYILGIINAFPA  GDINGDNVINSTDAVLLRRYLLLIIEEFPNPN-------GRLAADINRDGQINSTDYILLRRYILGIINEL--  GDLNGDGSIDSIDIVLFSRYLLDIIDEFPVDGD------ALRIADLNGDGYIDSSDYTLLRRYVLGVISEFPK  GDINGDGVIDSVDYVLIRRFILSIIDEFPNGQN------GFRAADINNDGRVDSADMVLLRRYLLGIINKLP-  GDLNRDGVIDSSDIVLLKRYILGIVDFDNN---------QLKYADLNLDNRVDSTDYNILKRYLLEIIPSIPL  GDLNGDGKVDSSDYVLLRRHILGVSELSST---------VLENADANSDGNIDSTDVILMRRYILGIIPTLG-  GDINNDGKINSVDCALMKRYLLEIIPFDE----------YTKHMDLNGDGLIDSSDYMLLKRYVLMITETFPA  **Type II Dockerins**  GDIVPDDAVNMTDIMKAIVAFNTTSGNDA----------FDEVADINKDGAVNMEDIMIIIRNFNRAPADYNR  GDIVYDTAINMNDIMKAVIAFNSVSGQEG----------YDEDADINKDNAVNMEDIMIIIKNFNRSSN | GH5_2-CBM6-DOC1  DOC1  DOC1  GH26-DOC1  GH74-DOC1  GH8-CBM11-DOC1  GH5_1-DOC1  GH18  DOC1  GH9-CBM3-DOC1  GH9-CBM3-CBM3-DOC1  GH11-CBM6-CE3-DOC1  GH43-CBM6-CBM6-DOC1  CBM35-GH26-DOC1  GH9-DOC1  GH9-CBM3-DOC1  CBM30-GH9-DOC1-CBM4-GH9  DOC1 peptidase_C1, BTB  CBM16-DOC1  CBM22-GH10-DOC1  DOC1 SCP  DOC1  GH5-CBM32-DOC1  GH5_2-CBM6-CBM6-DOC1  GH5_4-DOC1  DOC1  DOC1 Lamini_G_3, lpxD  GH44-DOC1-CBM44  CBM22-GH10-CBM22-DOC1  DOC1  GH53-DOC1  DOC1 alpha-2-macroglobulin  GH9-CBM3-DOC1  DOC1 alpha-2-macroglobulin  DOC1 peptidase_S8, Laminin  DOC1  DOC1  GH8-CBM6-DOC1  GH9-DOC1  GH9-CBM3-DOC1  GH5_1-DOC1  GH5_8-DOC1  GH9-CBM3-DOC1  GH5_1-DOC1  GH11-DOC1  DOC1  DOC1 peptidase_C1, BTB  CBM4-GH9-DOC1  GH81-DOC1  GH9-CBM3-CBM3-DOC1  GH16-CBM16-DOC1 Lamini_G  CBM30-GH9-DOC1  DOC1  GH9-CBM3-DOC1  DOC1  DOC1  DOC1  CBM30-GH9-DOC1  DOC1  GH48-DOC1  DOC1  DOC1  DOC1  GH5_34-CBM6-CBM13-CBM62-CBM6-DOC1  DOC1  GH10-GH62-CBM6-CBM6-DOC1  GH30_8-CBM6-DOC1  GH43-CBM13-DOC1  GH11-DOC1  DOC1 peptidase_S8, Laminin  CBM4-GH9-DOC1  CBM4-GH9-DOC1  GH16-DOC1  GH11-CBM6-GH11-CBM6-DOC1  DOC1 FN3, CYK3  DOC1  DOC1-EXPN-CBM63  DOC1  DOC1  DOC1  DOC1 RCC1, RhsA  DOC1 RCC1  DOC1 SCP  DOC1  DOC1  DOC1 Peptidase_S8, Peptidoglycan recognition  DOC1  DOC1  DOC1  DOC1-GH30  DOC1  DOC1  DOC1  DOC1 RCC1  DOC1-GH423  DOC1 RCC1, RshA  DOC1  DOC1 DOC1-CBM13 RicinB  DOC1  DOC1-EXPN-CBM63  DOC1 FN3, vWFA  DOC1  DOC1 serpin  DOC1 serpin  DOC1  DOC1  DOC1 serpin  DOC1  DOC1  DOC1  DOC1  DOC1 RshA, BTB, RHS Repeat  DOC1-CE4  DOC1  DOC1  DOC1  DOC1  DOC1-CBM6-GH10  CE1-CBM6-DOC1-GH10  DOC1  GH11-DOC1  DOC1  DOC1  GH11-CBM6-DOC1-CE15  GH11-CBM6-DOC1  DOC1  CE1-CBM6-DOC1-GH10  DOC1  DOC1 Leucine rich repeats  DOC1  DOC1 Peptidase_S8  DOC1-CBM6-PL1  CBM6-DOC1  DOC1  ScaA (scaffoldin)  ScaP2 (scaffoldin) |

**Multiple sequence alignment of the *C. alkalicellulosi* 136 dockerin modules.**

Cyan highlight indicates putative calcium-binding residues

Yellow highlight indicates putative recognition residues

**Abbreviations:**

**CBM** Carbohydrate binding module (followed by family number)

**CE** Carbohydrate esterase (followed by family number)

**COH** Cohesin

**DOC** Dockerin

**EXPN** Expansin

**FN3** Fibronectin type III domain

**GH** Glycoside hydrolase (followed by family number)
