# Supplementary material for: The Cellulosome Paradigm in An Extreme Alkaline Environment
Source: Microorganisms. 2019 Sep 12;7(9):347. doi: 10.3390/microorganisms7090347 (PMC6780208; doi:10.3390/microorganisms7090347)
Supplement: Supplementary file 1 [file microorganisms-07-00347-s001.zip › Table S3 .docx]

**Table S3.** Multiple sequence alignments of *C. alkalicellulosi* cohesin modules by ClustalO

**Type I and type II cohesin modules**

CloalDRAFT_1967 -----DKATASVGEIITAQVKISDIP---------QFAGYELNLIYDKNLIRPVKDD-LT

CloalDRAFT_2910-2 VSLSTDNHQPEVGDIITVSLDISNID---------NFAGYQANIIYDPLVIEPLDYDTEE

CloalDRAFT_3207 ----------KKGDIITASININEIS---------NLAGYQLNIKYDPKVLQPVDCITKN

CloalDRAFT_3961 -----DRTTAQKGEIIKVTVKVNNIK---------NLSGYQINIKYDPDILEMVNPNTGS

CloalDRAFT_0629 -----DKNSGEIGEIIKADIVLKSIN---------GIAGYQVNIKYDPKVLQAVNYDTGE

CloalDRAFT_3066-2 ITMELDKTFVKEGDIIRAAINISNIS---------NFAGYQLNIKFDPEVLQAVNPDTEV

CloalDRAFT_3067-1 IEMELDKTTANVGDIIKATIRIKDIT---------NFSGYQLNIKYDPEVLEAVNVETGE

CloalDRAFT_3067-2 IAMDLDKTLVEVGDIIKATIRIKDIT---------NFSGYQLNIKYDPEVLEAVNVETGE

CloalDRAFT_3067-3 IAMDLDKTLVEVGDIIKATIRIKDIT---------NFSGYQLNIKYDPEILEAVNVETGE

CloalDRAFT_0628-4 ISMVFDKKTASIGDIVTASININNIN---------NFAGYQVSIKYDPEVLEPINAVTGE

CloalDRAFT_0628-2 IELDLNQNVAQVGDIIRASVKVNNID---------NFAGYQVNIKYDPEVLQAVNPNTGE

CloalDRAFT_0628-3 IEMTFDKNTARVGEIIEASININNIS---------NFMGYQINIKYDPTVLEAVDTNTGV

CloalDRAFT_0628-1 IVLEVDKTNANVGDIILAKVNVYNID---------KLAGYQVNIKYDPEVLQAVDVDSGA

CloalDRAFT_3065 IYIDFDKTTVLKGDTISATIHIENIN---------KFAGFHVNLKYDPQVLQAIDLSSGK

CloalDRAFT_0274-2 INVNIGMVSGRPGQILEVPVYVSNIA---------PVKELKFALKLDPSTLDLISVDPSN

CloalDRAFT_20202 -HLNIGKVIGCPGDIVEIPVSISNVP---------PLAKLRFALNYDSCDLEIISVSPGE

CloalDRAFT_2910-1 -----PRVRGNPGETIQVPVHISNLP-------PEGISALSFGVNYDKRIFTYLGYEEGQ

CloalDRAFT_1780 ---------------IEIPISISNLP-------KEGISGFDFIVNYDNTKIKYTGFKETT

CloalDRAFT_0457 --LSVGDVVGMPRDIVEIPIELFNFT-------EEEIHSFHAKLTYNNNAIDVLSIKTGV

CloalDRAFT_0656 -------------DSVIVPITFTNVIHRDDPPSMGGIDNFEFVVEYDSNDLEAVEILPGS

CloalDRAFT_3567-2 -TVELEDISARKGETIEIPIYLKDLP-------DHGIDGLSFGLNYDPSKLDILSISDEG

CloalDRAFT_3500-2 --VEIEDVSAKIGETVLVPILLKDVP-------NDGIEQLYFDYIYDTTKLDLKYILNGE

CloalDRAFT_0274-1 VKISVKDERGTIGDVLKLSLELSDVP-------LTGICQLEFTLTYEQLELEILDISPSK

CloalDRAFT_2020-1 -SISIEDASGSTGDILTVSLRISNVP-------SQGLSECYFQVAFDGYDLEILDIVHGE

CloalDRAFT_3567-1 LVIQIGRASGFKNHIVEVPISFSNVP-------TCGLDHFEFKLKYELEDIEIIGIEPGD

CloalDRAFT_3500-1 LLVEIGTATGTKDHIVEIPINLSNVP-------LNGLEHFDFKLKYETEDIEILDLEPGE

CloalDRAFT_32901 ----IGTSEGSIWDQVEIPLTISGVP-------ESGINYIEFVLNYDSSDLDIITIIPGE

CloalDRAFT_4206 -----EFIQAKPGETVEVPVMMSNVP-------SRGINGLYLRLLYDNNLIDVTNIKPGD

CloalDRAFT_3064 FKVEIADVQGKQGDTVEVPIRFINVP-------EEGINSCDFTIGYDENVLELIQVKPGS

CloalDRAFT_3290-2 VFLQKERVLGEVGDIVEIPINLTDVS-------PNGVSHLDFLLDYNPSSLKIISLIPGE

CloalDRAFT_2305-2 ITLELETVKGNQGDIVKIPLNLTTMP-------TQGIEYLDFILGYDPDDLVILSIDAGD

CloalDRAFT_2305-3 ITIEIETVKGNPGDIVKIPLNLTTMP-------LKGINNVDFILRYNTDDLDILSIDAGD

CloalDRAFT_2305-1 IVLEVGTVVANPYDIVNIPIIIKDIP-------EDGISNLDFVLTYDTSCLNIISIEPGE

CloalDRAFT_3066-1 LNITVGLVEGKATETVAIPIYFENVP-------SGGINNADFVLSYDTSVINITEIVVGD

CloalDRAFT_3068-1 LTVTIGRVQGGTGTTVQIPVNFSGVP-------ASGINNCDFILTYNPSLLEVTDVTPGS

CloalDRAFT_3068-4 LTVTIGTVTASVGQTVQIPVYFSNVP-------ASGINNADFILTYNTSVLNVTDITAGA

CloalDRAFT_3068-2 LTVTIDSVTASVGQTVQIPVRFSNVP-------ASGINNADFILTYNTSVLNVTDITPGS

CloalDRAFT_3068-3 LTVTIDSVTASVGQTVQIPVYFSNVP-------ASGINNADFILTYNTSVLNVTDITPGA

CloalDRAFT_3068-5 LRVGIDTVLATAGQTVQIPVRFANVP-------SSGINNADFILWYDPAVLNVTDILPGS

CloalDRAFT_3068-6 --VIVNVVGGEAGQTVEIPVRLSNVP-------SSGINNADFILWYDPAVLNVTEIAPGA

CloalDRAFT_3068-9 --VIVNTVGGEAGQTVEIPITFSNVP-------TSGVNNADFILWYDADVLEVTEITAGA

CloalDRAFT_3068-7 --VIVNTVGGEAGQTVQIPVTLSNVP-------SSGINNADFILWYDADVLNVTEITPGA

CloalDRAFT_3068-8 --VIVNTVGGEAGQTVQIPVTLSNVP-------SSGINNADFILWYDADVLEVTEITAGA

CloalDRAFT_3068-10 --VIVNTVGGEAGQTVEIPVTFSKVP-------SSGINNADFVLWYDPAVLNVTEITAGA

: : . . . : :

CloalDRAFT_1967 PYTNRTMPSDRNVIINESFNPITFVNHDIERGYIG-FGVT--------YSSLEAY--RNA

CloalDRAFT_2910-2 QYSKDTMPKIGNILNST-YEYVEGAYHNLGEGRVN-FGRA--------VKSLSSY--KNS

CloalDRAFT_3207 PYEEDTIPSIGNVLSDTNHQYVSAAYHDLEEGRIN-FGRV--------VKSIDQF--INN

CloalDRAFT_3961 KTSD---GLIGNIITNSDFGILPVSSHNEDQGIFF-IGRS--------YLYLEDY--RAE

CloalDRAFT_0629 PLGNRTIPSGGTMIQNEAYNVLPLPGNNIEQGIIN-FGKT--------YVNLNAY--KES

CloalDRAFT_3066-2 AFARRTNPLPGTILVNDEYGNLLVAVNVIGDGILN-FGNT--------YSDLAKY--RAS

CloalDRAFT_3067-1 AYDDSTMPQSGDILANPDLGNVTAVSHSTSEGILN-FGNA--------YQYLDDY--IEL

CloalDRAFT_3067-2 AYDDSTMPQSGDILANPDLGNVTAVSHSTSEGILN-FGNA--------YQYLEDY--IEI

CloalDRAFT_3067-3 AYDDSTMPQSGDILSNPDLGNVTAVSHSTSEGILN-FGNA--------YQYLEDY--IEL

CloalDRAFT_0628-4 AYGRRTMPESGSILVNSTFSPLTVASHDLSEGRLS-FGKT--------YTELSAY--RTS

CloalDRAFT_0628-2 VLSIRSMPLDGNILVNSEYGVISAANNDIQRGLLN-FAKS--------YTYLDDY--KNS

CloalDRAFT_0628-3 AFGRRTMPANGTILVNENYGVIEAVNNNIDEGIIN-FAKT--------YSNAEQY--KLN

CloalDRAFT_0628-1 AFTSNTKPKNGNLINNADLGIIDAVSNDVERGILN-FGRT--------YTYLDDY--RGL

CloalDRAFT_3065 PYDSDTLPEGGTVIVNMDYYPISAVSNDISIGQIN-FGRT--------YTLLKEY—KER

CloalDRAFT_0274-2 IISD------------IEGSLV--YMRSFSRGEVGPDGNSITTHLACNLQYFEFYYVEHD

CloalDRAFT_20202 AIPD------------SEFVDF-SYELDIEEFPV-LYGQG------IIFNYSEKNQHLRD

CloalDRAFT_2910-1 LTDS-------------SDA---YIEANEGNSGVR-------------VF-YSDNTNTYT

CloalDRAFT_1780 ITNS-------------IFA---NIEAVEISKGVK-------------VL-YIDESEEGN

CloalDRAFT_0457 AMYT--------------SQSF-SCKFDNSDGSII-------------LS-FEKQS--EY

CloalDRAFT_0656 ILPN------------IEDTNF-SYEIRPEDNEIS-------------FK-FKVNEADIY

CloalDRAFT_3567-2 LVSI--------------FY------YYIFEGSIG-------------FN-FLSL----N

CloalDRAFT_3500-2 LLED--------------DIRF-VSGIDRTNGKIG-------------FY-VMKS----N

CloalDRAFT_0274-1 IIEN-------------ADENF-EVSILDIGSSIV-------------FS-FEDKT-KGS

CloalDRAFT_2020-1 IVEN-------------EDN-F-NW-YVSENSVII-------------FS-FNDKT-EGN

CloalDRAFT_3567-1 IVMN-------------PEKDL-GASLNYDESSIL-------------FV-FTDESAVGK

CloalDRAFT_3500-1 IIRN-------------PETDF-AVSINANTGILI-------------FC-FSDMSAIAE

CloalDRAFT_32901 ILTN-------------PLETF-AIGIDY--NSIV-------------IM-FINDTG--I

CloalDRAFT_4206 IIND-------------PDKDF-AYNVAENTGTIV-------------IL-YTEDSQTGK

CloalDRAFT_3064 IIKN-------------PNSNF-SYNSPK-PGRTV-------------FM-FTDETGHGR

CloalDRAFT_3290-2 IVPD-------------PNFTF-AWSLNENNGHIS-------------VM-FCDN-----

CloalDRAFT_2305-2 IIEN-------------SHINF-ASDIKK-DGRLD-------------FM-YTDESGEGT

CloalDRAFT_2305-3 IIEN-------------ASLNF-ASQINENKGLLS-------------FM-FTDESGTSA

CloalDRAFT_2305-1 ILKD-------------HSTDF-VTNINSNTGRMV-------------FM-FTDETGTGE

CloalDRAFT_3066-1 IITN--------------SANF-SSSVNSEEGTIS-------------FL-FIDETGLGL

CloalDRAFT_3068-1 IIQN-------------GSTDF-ASNINGEDGRIS-------------FM-FTDETGRGQ

CloalDRAFT_3068-4 IIQN-------------GDTDF-ASNINDSAGRLS-------------FM-FTDETGRGQ

CloalDRAFT_3068-2 IIQN-------------GDTDF-AFNINADAGRLS-------------FM-FTDETGRGQ

CloalDRAFT_3068-3 IIQN-------------GDTDF-AFNINADGGRLS-------------FM-FTDETGRGQ

CloalDRAFT_3068-5 IIAN-------------GETDF-AFNINEETGRLS-------------FM-FTDETGRGQ

CloalDRAFT_3068-6 IIEG-------------GSTDF-ASNINEETGRLS-------------FM-FTDETGRGQ

CloalDRAFT_3068-9 IIEG-------------GSTDF-ASNINEETGRLS-------------FM-FTDETGRGQ

CloalDRAFT_3068-7 IIEG-------------GSTDF-ASNINEETGRLS-------------FM-FTDETGRGQ

CloalDRAFT_3068-8 IIEG-------------GSTDF-ASNINEETGRLS-------------FM-FTDETGRGQ

CloalDRAFT_3068-10 IIEG-------------GSTDF-ASNINEETGRLS-------------FM-FTDETGRGQ

CloalDRAFT_1967 GIIETGDTLGIIKFEVLEE----NLIEIKVED--FKLP---------SSNGVKFFDVYGS

CloalDRAFT_2910-2 GSAETTGNLAKIKFRILRE----VESTNIKLDDYTNDIYERAEGAIIKQRDIQLFDWDGN

CloalDRAFT_3207 ESIVKTGELAKISFEVLKL----KDTKIEFIN----------------------------

CloalDRAFT_3961 NMPEDTGILFEVGFKVLKE----EMTSIEFTN--INLP--------NAISGTVLFDWHGN

CloalDRAFT_0629 GSLEKEGTFATIGFKILEK----TSTSIRFED-IGTMP--------TAISGTMLFDLNGE

CloalDRAFT_3066-2 GNPEETGTIAIIGFKVLQE----KDTEIKFEDDLEKMP--------GAISGTFLFDWNGE

CloalDRAFT_3067-1 EEPEETGVIAVIGFKVLQA----KDTEIKFVD-TDSMP--------NAITGTQLFDWESE

CloalDRAFT_3067-2 NEPEETGVIAVIGFKVLQA----KDTEIKFVD-TDSMP--------NAITGTQLFDWESE

CloalDRAFT_3067-3 GEPEETGVIAVIGFKVLQA----KDTEIKFVD-TDSMP--------NAITGTQLFDWESE

CloalDRAFT_0628-4 GTGENSGTLANISFRVLKT----EKTLVSFEE-TSASG--------ASIFGTLLYDWNGY

CloalDRAFT_0628-2 GDPHSTGIIAEIGFKAINI----GNTNIVFED-TSTMM--------SGIQGTMLYNWNGE

CloalDRAFT_0628-3 GGAEESGTIGVIGFRVLKE----ESTSVSFEN-TVTMP--------GGLTGTLLFNWDGN

CloalDRAFT_0628-1 GDVEEIGTLAVIGFKVLEQ----KPTSIRFEN-TPTMP--------TGVIGTMLFNWDGD

CloalDRAFT_3065 GQAEESGILARIGFKVLQE----KDTKIIFEN-SSAMP--------YGISGTMLFEWDGN

CloalDRAFT_0274-2 LEMLYDIKIATITVEIKKDVNGKEFSPIEYID-LSYDG--------------PANDEFYS

CloalDRAFT_20202 QQLLSDGKFAIITAKIKA-STNKKFRPIRYVN-PSYME----------------------

CloalDRAFT_2910-1 APLVDDGELIKLNFQINSQ-AQESAYTLAYL-----PG--------------WSFTKVMP

CloalDRAFT_1780 QPLDRSGEIIKLIFELDSS-FD-GICQVSFS-----DG--------------WCFSKIGE

CloalDRAFT_0457 DYIQEDSEMVTIVARIKTD-KYGNCNIDTS----SDIA--------------PIFKTS--

CloalDRAFT_0656 DLIRQDGDFASIKFNLKDP-SSKEFYPISI------------------------------

CloalDRAFT_3567-2 DKITKDGILAFVTVRVKED-ASVGSTQII-------------------------------

CloalDRAFT_3500-2 NKITEDGVFAYLKFRIKEN-LGFESTEI--------------------------------

CloalDRAFT_0274-1 ELIRTDGEFAQITVKIKAG-AFNGPSIFHLN---SIRQ--------------VNFNNA--

CloalDRAFT_2020-1 ELIKSNGEFAKITLRIKPT-ALKGSSFFRLG---G-------------------------

CloalDRAFT_3567-1 RNIKNDGVFATIIIRVKNE-SEK-FSLIE----LLNQR--------------PLFSNY--

CloalDRAFT_3500-1 RTINDDGILATLIARIKNE-DET-FSAITK---LTNSN--------------PIFGDY--

CloalDRAFT_32901 DSIKDDGVLLKIVATIRNE-HKLDLSEISL-----KSF--------------GTIILS--

CloalDRAFT_4206 GMIDTDGVFMTLEVKVKDN-ALSGTDSVYAA--ISVDS--------------SSVSDF--

CloalDRAFT_3064 EIINSDGEFARVTFKIKTS-SKEQSSAIIP------DK--------------WTFANY--

CloalDRAFT_3290-2 -IILEDGEFVKITAEILHN-SQAKLNEITL-----WSY--------------TSNQV---

CloalDRAFT_2305-2 YKINELGVFATLTVMIKDE-AKTGLSEVVL-----LNV--------------GNIGY---

CloalDRAFT_2305-3 YNIRELGEYAIITARIKEE-AQLGLSEIVL-----HSI--------------APTPW---

CloalDRAFT_2305-1 RKITEEGEFASITARVSLS-AKEGYSEINL-----GV-----------------------

CloalDRAFT_3066-1 EQINLDGTFAKIFASIKED-APSGLSVIEF-----VYE--------------TTVTDM--

CloalDRAFT_3068-1 RMIQTDGTFATITARVISN-SPA---TVSL-----AQR--------------GAIADY--

CloalDRAFT_3068-4 RMITADGVFATITATVRTN-ARG---PISL-----AQQ--------------GAIADY--

CloalDRAFT_3068-2 RMITTDGVFATITATVRTN-ARG---PIAL-----AQQ--------------GAIADY--

CloalDRAFT_3068-3 RMITTDGVFATITATVRTN-ARG---PISL-----AQQ--------------GAIADY--

CloalDRAFT_3068-5 RMITADGVFATITATVRTT-ATSGVSEITL-----AQT--------------GAIADY--

CloalDRAFT_3068-6 RMIKADGVFATITATISAS-APNGFSAIRL-----AQQ--------------GAIADY--

CloalDRAFT_3068-9 RMIKADGVFATITATISAS-APNGLSEIKL-----ASQ--------------GAIADY--

CloalDRAFT_3068-7 RMIKADGVFATITATISAS-APNGLSEIKL-----AQQ--------------GAIADY--

CloalDRAFT_3068-8 RMIKADGVFATITATISAS-APNGLSEIKL-----AQQ--------------GAIADY--

CloalDRAFT_3068-10 RMIKSDGVFATITATISAS-APNGLSEIKL-----ASQ--------------GAIADY--

:

CloalDRAFT_1967 EI-----------------------

CloalDRAFT_2910-2 -------------------------

CloalDRAFT_3207 -------------------------

CloalDRAFT_3961 VI-----------------------

CloalDRAFT_0629 RIS----------------------

CloalDRAFT_3066-2 KLLDYVVIQPLPIKA----------

CloalDRAFT_3067-1 TVTGYKVIQPEPIK-----------

CloalDRAFT_3067-2 TVTGYKVIQPE--------------

CloalDRAFT_3067-3 TIFGYRVIQPE--------------

CloalDRAFT_0628-4 RLSNYSVLQPGELN-----------

CloalDRAFT_0628-2 RISGYNV------------------

CloalDRAFT_0628-3 QVLNYSVIQPQTI------------

CloalDRAFT_0628-1 VIANYSVVQP---------------

CloalDRAFT_3065 RVSDYKVIQPE--------------

CloalDRAFT_0274-2 TKISPKKLELQFINGGVHI------

CloalDRAFT_20202 -------------------------

CloalDRAFT_2910-1 NGSVVEEENVLFKIG----------

CloalDRAFT_1780 DKEVYEIENVIFRSG----------

CloalDRAFT_0457 ---TMSDMTTKFNRGLVK-------

CloalDRAFT_0656 -------------------------

CloalDRAFT_3567-2 -------------------------

CloalDRAFT_3500-2 -------------------------

CloalDRAFT_0274-1 ---DVEIYEPYFLTKALQVG-----

CloalDRAFT_2020-1 -------------------------

CloalDRAFT_3567-1 ---DYTMMPFVFKSGGVNL------

CloalDRAFT_3500-1 ---DYTYYPFVFKSGGVKI------

CloalDRAFT_32901 ---EPSFVNPVFSIGGVHIIESAP-

CloalDRAFT_4206 ---DLKEI-----------------

CloalDRAFT_3064 ---SLKSFSTEFARGSVRV------

CloalDRAFT_3290-2 ----SLSNCSAFITGGIII------

CloalDRAFT_2305-2 ----LKPIGLEFINGGV--------

CloalDRAFT_2305-3 ----FCPIRLEFINGGVEIT-----

CloalDRAFT_2305-1 -----NYERFTFIKGGV--------

CloalDRAFT_3066-1 ---DLNPINFVLNNGGVNVKKSEQT

CloalDRAFT_3068-1 ---DLSTISATFVDGGVNLGST---

CloalDRAFT_3068-4 ---DLRTIPATFVDGGVNLGST---

CloalDRAFT_3068-2 ---DLRTISATFVDGGVNLGST---

CloalDRAFT_3068-3 ---DLRTISATFVDGGVNLGST---

CloalDRAFT_3068-5 ---DLRNIPSAFTNGGVNVGGSI--

CloalDRAFT_3068-6 ---DLRTIPATFVDGGVNVG-----

CloalDRAFT_3068-9 ---DLKTIPATFVDGGVIVG-----

CloalDRAFT_3068-7 ---DLKTIPATFVDGGVNVG-----

CloalDRAFT_3068-8 ---DLKTIPATFVDGGVNVG-----

CloalDRAFT_3068-10 ---DLKTIPATFVDGGVDVGSTV--

Green highlight indicates type II cohesin modules.

No highlight indicates type I cohesin modules.
