# Supplementary material for: The Cellulosome Paradigm in An Extreme Alkaline Environment
Source: Microorganisms. 2019 Sep 12;7(9):347. doi: 10.3390/microorganisms7090347 (PMC6780208; doi:10.3390/microorganisms7090347)
Supplement: Supplementary file 1 [file microorganisms-07-00347-s001.zip › Table S5 .docx]

**Table S5.** Cross-species interactions between *C. alkalicellulosi* cohesins and dockerins and those of other species.

| Cohesins |  |  |  |  |  |  |  |  |
| --- | --- | --- | --- | --- | --- | --- | --- | --- |
|  | *Ct*Doc1 | | *Ac*Doc1 | *Bc*Doc1 | *Af*Doc1 | *Cc*Doc1 | *Rf*Doc1 | *Ca*ScaA(XDoc2) |
| *Ca*ScaA(Coh1) | +++ | | – | + | – | – | – |  |
| *Ca*ScaD(Coh1) | + | | – | – | – | – | – |  |
| *Ca*ScaG(Coh1) | + | | – | – | – | – | – |  |
| *Ct*ScaB(Coh2) |  | |  |  |  |  |  | – |
| *Ct*ScaC(Coh2) |  | |  |  |  |  |  | – |
| *Ct*ScaF(Coh2) |  | |  |  |  |  |  | +++ |

>0.5 = +++, 0.3-0.5 = ++, 0.1-0.3 = +

| *Ac – A. cellulolyticus*  *Af – A. fulgidis*  *Bc – B. cellulosolvens*  *Ca – C. alkalicellulosi*  *Cc – C. cellulolyticum*  *Ct – C. thermocellum*  *Rf – R. flavefaciens* | Coh1 – type I cohesin  Coh2 – type II cohesin  Doc1 – type I dockerin  Doc2 – type II dockerin |
| --- | --- |

*Ca*ScaA(Coh1) – type I cohesin from *C. alkalicellulosi* ScaA (CloalDRAFT_3068) [this publication]

*Ca*ScaD(Coh1) – type I cohesin from *C. alkalicellulosi* ScaD (CloalDRAFT_3064) ) [this publication]

*Ca*ScaG(Coh1) – type I cohesin from *C. alkalicellulosi* ScaG (CloalDRAFT_4206) ) [this publication]

*Ca*ScaA(XDoc2) – type II X-dockerin dyad from *C. alkalicellulosi* ScaA (CloalDRAFT_3068) ) [this publication]

*Ct*ScaB(Coh2) – type II cohesin from *C. thermocellum* ScaB (OlpB) [1]

*Ct*ScaC(Coh2) – type II cohesin from *C. thermocellum* ScaC (Orf2p) [1]

*Ct*ScaF(Coh2) – type II cohesin from *C. thermocellum* ScaF (SdbA) [1]

Type I dockerins were derived from the respective GH48 enzyme of the given species [1-7], except for the type I dockerin of *B. cellulosolvens* which was derived from ScaA

**References**

1. Yoav, S., Barak, Y., Shamshoum, M., Borovok, I., Lamed, R., Dassa, B., Hadar, Y., Morag, E., and Bayer, E. A. (2017) How does cellulosome composition influence deconstruction of lignocellulosic substrates in *Clostridium (Ruminiclostridium) thermocellum* DSM 1313. *Biotechnol. Biofuels* *10:222*.
2. Pagès, S., Belaich, A., Belaich, J.-P., Morag, E., Lamed, R., Shoham, Y., and Bayer, E. A. (1997) Species-specificity of the cohesin-dockerin interaction between *Clostridium thermocellum* and *Clostridium* *cellulolyticum:* Prediction of specificity determinants of the dockerin domain. *Proteins* *29*, 517-527.
3. Hamberg, Y., Ruimy-Israeli, V., Dassa, B., Barak, Y., Lamed, R., Cameron, K., Fontes, C. M., Bayer, E. A., and Fried, D. B. (2014) Elaborate cellulosome architecture of *Acetivibrio cellulolyticus* revealed by selective screening of cohesin-dockerin interactions *PeerJ* *2:e636*.
4. Zhivin, O., Dassa, B., Moraïs, S., Uttukar, S. M., Brown, S. D., Henrissat, B., Lamed, R., and Bayer, E. A. (2017) Unique organization and unprecedented diversity of the *Bacteroides (Pseudobacteroides) cellulosolvens* cellulosome system. *Biotechnol. Biofuels* *10:211*.
5. Voronov-Goldman, M., Lamed, R., Noach, I., Borovok, I., Kwiat, M., Rosenheck, S., Shimon, L. J. W., Bayer, E. A., and Frolow, F. (2011) Non-cellulosomal cohesin from the hyper-thermophilic archaeon *Archaeoglobus fulgidus*. *Proteins* *79*, 50-60.
6. Reverbel-Leroy, C., Pagés, S., Belaich, A., Belaich, J.-P., and Tardif, C. (1997) The processive endocellulase CelF, a major component of the *Clostridium cellulolyticum* cellulosome: Purification and characterization of the recombinant form. *J. Bacteriol.* *179*, 46-52.
7. Israeli-Ruimy, V., Bule, P., Jindou, S., Dassa, B., Barak, Y., Slutzki, M., Hamberg, Y., Cardoso, V., Alves, V. D., Najmudin, S., White, B. A., Flint, H. J., Gilbert, H. J., Lamed, R., Fontes, C. M. G. A., and Bayer, E. A. (2017) Complexity of the *Ruminococcus flavefaciens* FD-1 cellulosome reflects an expansion of family-related protein-protein interactions. *Sci. Rep.* *7*, 42355.
